# Novel Diterpenoids from the Twigs of *Podocarpus nagi*

**DOI:** 10.3390/molecules21101282

**Published:** 2016-09-24

**Authors:** Yuan-Dong Zheng, Xing-Chen Guan, Dan Li, An-Qi Wang, Chang-Qiang Ke, Chun-Ping Tang, Li-Gen Lin, Yang Ye, Zheng-Liang Wang, Sheng Yao

**Affiliations:** 1 State Key Laboratory of Drug Research & Natural Products Chemistry Department, Shanghai Institute of Materia Medica, Chinese Academy of Sciences, Shanghai 201203, China; zhengyd@shanghaitech.edu.cn (Y.-D.Z.); kechangqiang@simm.ac.cn (C.-Q.K.); tangcp_sh@163.com (C.-P.T.); yye@simm.ac.cn (Y.Y.); 2 University of Chinese Academy of Sciences, Beijing 100049, China; 3 School of Life Science and Technology, ShanghaiTech University, Shanghai 201203, China; 4 School of Chemistry & Environmental Engineering, Yangtze University, Jingzhou 434023, China; gxc911212@126.com; 5 State Key Laboratory of Quality Research in Chinese Medicine, Institute of Chinese Medical Sciences, University of Macau, Macau 999078, China; yb47516@umac.mo (D.L.); yb37510@umac.mo (A.-Q.W.); ligenl@umac.mo (L.-G.L.)

**Keywords:** *Podocarpus nagi*, abietane-type diterpenoid, *ent*-pimarane-type diterpenoid, cytotoxicity, anti-inflammation

## Abstract

Phytochemical investigation of the twigs of *Podocarpus nagi* (Podocarpaceae) led to the isolation of two new abietane-type diterpenoids, named 1β,16-dihydroxylambertic acid (**1**) and 3β,16-dihydroxylambertic acid (**2**), along with two new *ent*-pimarane-type diterpenoids, named *ent*-2β,15,16,18-tetrahydroxypimar-8(14)-ene (**3**) and *ent*-15-oxo-2β,16,18-trihydroxypimar-8(14)-ene (**4**). Their respective structures were elucidated on the basis of spectroscopic analyses, including 1D- and 2D-NMR, IR, CD, and HR-ESI-MS. This is the first time *ent*-pimarane-type diterpenoids from the genus *Podocarpus* has been reported. All four new compounds were tested for cytotoxic activity. The MTT assay results showed that compounds **3** and **4** significantly inhibited the proliferation of human cervical cancer Hela cells, human lung cancer A549 cells, and human breast cancer MCF-7 cells at a concentration of 10 μM. Furthermore, using the lipopolysaccharide (LPS)-stimulated RAW264.7 cells, compounds **2** and **4** were found to significantly inhibit nitrogen oxide (NO) production with IC_50_ values of 26.5 ± 6.1 and 17.1 ± 1.5 μM, respectively.

## 1. Introduction

**Figure 1:**
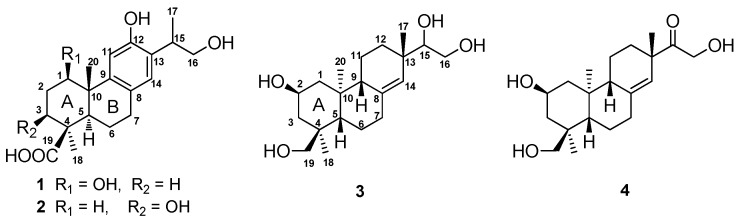
Chemical structures of compounds **1**–**4**.

*Podocarpus nagi* (Thunb) Zoll. et Mor ex Zoll (Podocarpaceae) is one of the most ancient Gymnosperms widely distributed in East Asia and the Southern Hemisphere [1]. As a traditional herbal medicine, the leaves and roots of *P. nagi* have been used for the treatment of rheumatism and arthritis, as well as venereal diseases [2]. A number of diterpenoids [3,4,5,6] and flavonoids [7] have been identified from this plant in previous investigations, and some of them exhibited cytotoxic [4,7,8,9], insecticidal [10], and antifungal [3] properties. Previously, we identified two cyclopeptides from the stem barks of *P*. *nagi* [11], and found that nagilactone B, a major diterpenoid from this plant, suppressed atherosclerosis in apoE deficient mice [12]. In our ongoing research, two new abietane-type diterpenoids and two new *ent*-pimarane-type diterpenoids were identified from the twigs of *P*. *nagi*. (Figure 1). Herein, details of the isolation and structure elucidation of these compounds, as well as their cytotoxic and anti-inflammatory activities are described.

## 2. Results

Compound **1** was obtained as a white amorphous powder. The molecular composition of **1**, C_20_H_28_O_5_, was deduced from the positive ion peak at *m*/*z* 349.2001 [M + H]^+^ (calcd. 349.2010) in its HR-ESI-MS, with seven degrees of unsaturation. The IR spectrum exhibited the absorptions at 3430, 2932, 1696, 1616, and 1418 cm^−1^, indicating the presence of hydroxyl, an aromatic ring, and carboxyl functionalities. The ^1^H-NMR spectrum of **1** exhibited the signals of three methyl groups (δ_H_ 1.13, s; 1.22, d, *J* = 6.8 Hz; 1.24, s), one hydroxyl methyl group (δ_H_ 3.48, dd, *J* = 10.2, 7.4 Hz; 3.69, dd, *J* = 10.2, 5.7 Hz), one oxymethine group (δ_H_ 3.52, dd, *J* = 11.7, 4.9 Hz), and two aromatic protons (δ_H_ 6.36, s; 8.05, s) (Table 1 and Supplementary Figure S2). The ^13^C-NMR spectrum of **1** displayed 20 carbons signals attributed to one carbonyl carbon (δ_C_ 181.2), six aromatic carbons (δ_C_ 153.7, 140.0, 136.1, 130.0, 128.9, and 115.5), two quaternary carbons (δ_C_ 45.0 and 44.3), three methine carbons (δ_C_ 79.1, 53.9, and 37.6), five methylene carbons (δ_C_ 68.4, 37.0, 33.5, 31.5, and 22.4), and three methyl carbons (δ_C_ 29.1, 18.2, and 17.3) (Table 1 and Supplementary Figure S3). The ^1^H- and ^13^C-NMR data of **1** were quite similar to those of the aglycone of 19-*O*-d-glucopyranoside of 16-hydroxylambertic acid except that a methylene group in ring A of the aglycone of 19-*O*-d-glucopyranoside of 16-hydroxylambertic acid was replaced by an oxymethine group in compound **1**, as well as the down-field shift of an aromatic acid (8.05 in **1**; 6.54 in 19-*O*-d-glucopyranoside of 16-hydroxylambertic acid) [13].

**Figure 2:**
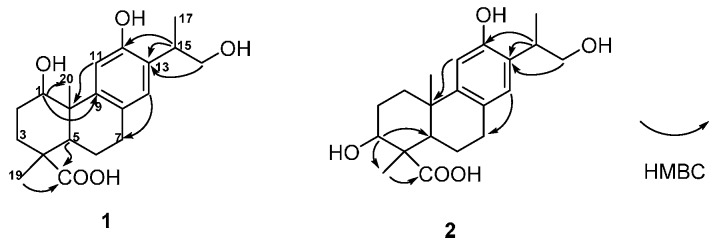
Key HMBC correlations of compounds **1** and **2**.

**Figure 3:**
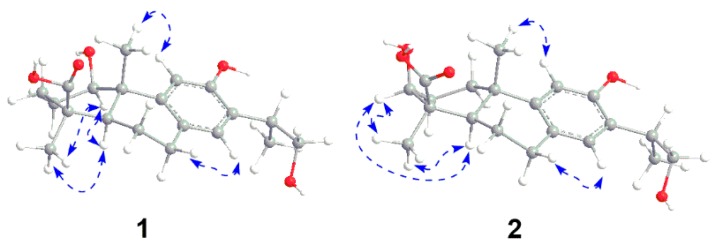
Key NOESY correlations of compounds **1** and **2**.

The structure of **1** was constructed by the detailed analysis of the HSQC and HMBC spectra (Figure 2 and Supplementary Figure S4 and S5). The correlations from H-11 to C-10, from H-14 to C-7, from H-15 to C-12, C-13, and from H_2_-16 to C-13 manifested a hydroxyl group on C-12, as well as a hydroxyl-substituted isopropyl substituent on C-13. Furthermore, the HMBC cross-peaks from H-1 to C-9 and C-20 suggested a hydroxyl group was substituted on C-1. The down-field shift of H-11 also supported a hydroxyl group was substituted on C-1 [14,15]. In addition, the correlated signals of H_3_-18 and H-5 to C-19 confirmed the carboxyl group on C-19. The relative configuration of **1** was subsequently deduced from the NOESY experiment. The hydroxyl group on C-1 was assigned as *β*-oriented based on the correlations from H_3_-18 to H-1 and H-5, as well as H-5 to H-1 (Figure 3 and Supplementary Figure S6). The absolute configuration of compound **1** was determined by CD spectrum. The Cotton effect of compound **1** (Δε_211_ 4.14, Δε_227_ 3.91, Δε_265_ −2.99) was consistent with that of ferruginol (Δε_211_ 4.11, Δε_227_ 3.97, and Δε_265_ −2.95), indicating a 10*S* configuration [16]. Thus, the structure of **1** was established as shown in Figure 1, and it was named 1β,16-dihydroxylambertic acid.

Compound **2** was isolated as a white amorphous powder. The molecular formula was determined to be C_20_H_28_O_5_ from the ion peak at *m/z* 349.2004 [M + H]^+^ (calcd. 349.2010) in its HR-ESI-MS. The IR spectrum of **2** displayed the absorptions for hydroxyl groups (3530 and 3385 cm^−1^), an aromatic ring (1616 and 1383 cm^−1^), and a carboxyl group (1688 cm^−1^). The ^1^H- and ^13^C-NMR spectra of **2** were quite similar to those of the aglycone of the 19-*O*-d-glucopyranoside of 16-hydroxylambertic acid except that a methylene group in ring A of the aglycone of the 19-*O*-d-glucopyranoside of 16-hydroxylambertic acid was replaced by an oxymethine group in compound **2**, which inferred a hydroxyl group might substitute at ring A [13]. The hydroxyl group was assigned on C-3 based on the HMBC correlations from H-3 to C-5 and C-18 (Figure 2). The NOE cross-peaks from H_3_-18 to H-3 and H-5, and H-5 to H-3 indicated the hydroxyl group was β-oriented (Figure 3). Moreover, the identical Cotton effect of compounds **2** and **1** suggested they shared the same absolute configuration at C-10. Thus, the structure of **2** was determined as shown in Figure 1, and it was named 3β,16-dihydroxylambertic acid.

**Figure 4:**
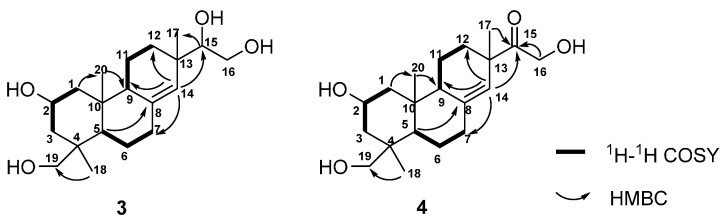
Key ^1^H-^1^H COSY and HMBC correlations of compounds **3** and **4**.

**Figure 5:**
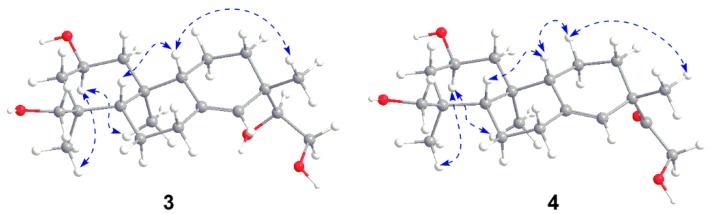
Key NOESY correlations of compounds **3** and **4**.

Compound **3** was obtained as a white amorphous powder. The molecular formula of **3**, C_20_H_34_O_4_, was deduced by the ion peak at *m*/*z* 383.2438 [M + HCOO]^−^ (calcd. 383.2439) in its HR-ESI-MS. The existence of hydroxyl group and double bond was deduced by the IR absorptions at 3424 and 1647 cm^−1^, respectively. The ^1^H-NMR spectrum of **3** showed the signals of three methyl groups (δ_H_ 0.85, s; 0.90, s; 0.99, s), two oxygenated methylenes (δ_H_ 3.05, d, *J* = 11.0 Hz, 3.36, m; 3.38, m, 3.74, dd, *J* = 11.1, 2.4 Hz), two oxygenated methines (δ_H_ 3.34, m; 3.85, m), and an olefinic proton (δ_H_ 5.37, s) (Table 2). The ^13^C-NMR spectrum revealed 20 carbon signals attributed to three methyl carbons, eight methylene carbons, four *sp*^3^ methine carbons, three *sp*^3^ quaternary carbons, one *sp*^2^ methine carbon, and one *sp*^2^ quaternary carbon (Table 2). The ^1^H- and ^13^C-NMR data of **3** quite resembled to those of kirenol except for the chemical shifts of H_3_-18, C-18, and C-19 [17,18]. Detailed analysis of the ^1^H-^1^H COSY, HSQC, and HMBC spectra resulted in the construction of planar structure of **3** (Figure 4). Subsequently, 2-hydroxyl group and H_3_-18 were assigned as β-oriented and α-oriented, respectively, by the correlations from H_3_-20 to H-2, and H-2 to H_3_-18 in the NOESY spectrum (Figure 5). The correlations from H-5 to H-9, together with H-9 to H_3_-17 indeed confirmed the two protons and H_3_-17 were *β*-oriented. Thus, the structure of **3** was elucidated as *ent*-2β,15,16,18-tetrahydroxypimar-8(14)-ene (Figure 1).

Compound **4** was obtained as a white amorphous powder. The molecular formula was determined to be C_20_H_28_O_5_ from the ion peak at *m*/*z* 337.2374 [M + H]^+^ (calcd. 337.2373) in its HR-ESI-MS, suggesting the presence of five degrees of unsaturation. The IR spectrum showed absorptions for a double bond at 1618 cm^−1^, a ketone carbonyl group at 1710 cm^−1^ and hydroxyl groups at 3426 cm^−1^. The ^1^H- and ^13^C-NMR spectra of **4** were quite similar to those of compound **3** (Table 2). After careful comparison, the chemical shifts of C-15 (80.8 ppm in **3**; 215.3 ppm in **4**) and H_2_-16 (3.38 and 3.74 ppm in **3**; 4.36 and 4.32 ppm in **4**) were obviously downfield shifted, implying that a ketone group in **4** might replace the hydroxyl group on C-15 in **3**. The HMBC correlations from H-14, H-16, and H_3_-17 to the carbonyl carbon assigned it as C-15 (Figure 4). The NOESY experiment further confirmed the relative configuration of **4** (Figure 5). Thus, the structure of **4** was established as *ent*-15-oxo-2β,16,18-trihydroxypimar-8(14)-ene as shown in Figure 1.

All the new compounds were tested for their cytotoxic activity on human cervical cancer Hela cells, human lung cancer A549 cells, and human breast cancer MCT-7 cells. The MTT assay results showed compounds **3** and **4** significantly inhibited proliferation on three cell lines at the concentration of 10 μM. The inhibitory rates for compounds **3** and **4** were 60.7% ± 1.9% and 62.6% ± 1.6% on Hela cells, 26.4% ± 4.1% and 29.0% ± 1.1% on A549 cells, and 41.2% ± 1.4% and 44.7% ± 1.9% on MCF-7 cells, respectively. However, compounds **1** and **2** did not show obvious cytotoxicity at 20 μM against the three cell lines. Herein, paclitaxel was used as the positive control, and the inhibitory rate of paclitaxel (200 nM) on Hela, A549, and MCF-7 cells were 56.2% ± 5.4%, 66.7% ± 8.8% and 43.3% ± 8.1%, respectively.

Additionally, the new compounds were evaluated for their inhibitory effect on NO production in LPS-stimulated RAW264.7 cells. The results showed compounds **2** and **4** significantly reduced NO production, with IC_50_ values of 26.5 ± 6.1 and 17.1 ± 1.5 μM, respectively, which were comparable with that of the positive control indomethacin (IC_50_ 4.5 ± 0.2 μM). The other two compounds did not show any inhibitory effect up to 100 μM. On the other hand, compounds **1**–**4** did not show obvious cytotoxicity at 100 μM against RAW264.7 cells.

## 3. Materials and Methods

### 3.1. General Experiments

TLC was performed on pre-coated silica gel GF254 plates (Merck Chemical Co. Ltd., Shanghai, China). MCI (Polystyrene) gel (CHP20P, 75−150 μm, Mitsubishi Chemical Industries, Tokyo, Japan), silica gel (Qingdao Marine Chemical Industrials, Qingdao, Shandong, China), macro porous resin AB-8 (Shandong Lu Kang Chemical Industrials, Jinan, Shandong, China), and Sephadex LH-20 (Pharmacia Biotech AB, Uppsala, Sweden) were used for column chromatography (CC). Analytical HPLC was applied on a Waters 2695 instrument (Milford, MD, USA) coupled with a 2998 PDA, a Waters 2424 ELSD, and a Waters 3100 MS detector. Preparative HPLC was performed on a Varian PrepStar pumps with an Alltech 3300 ELSD (Columbia, MD, USA) using a Waters Sunfire RP C18, 5 μm, 30 × 150 mm column. Optical rotations were measured on a Rudolph Autopol VI Automatic polarimeter (Hackettstown, NJ, USA). IR data were recorded on a Nicolet Magna FTIR-750 spectrophotometer (Waltham, MA, USA). NMR spectra were recorded on a Bruker Avance III (Bruker, Zurich, Switzerland) for 500M and 600M NMR spectrometer and a Varian MR-400 (Varian, Palo Alto, CA, USA) for 400M NMR spectrometer with TMS as the internal standard. CD spectra were measured on a Jasco J-180 spectrophotometer (Mitsubishi Chemical Industries, Tokyo, Japan). HR-ESI-MS were measured on a Waters Xevo Q-Tof mass detector and an Agilent G6520 Q-TOF mass detector (Santa Clara, CA, USA). All solvents used for CC and HPLC were of analytical grade (Shanghai Chemical Reagents Co. Ltd., Shanghai, China) and gradient grade (Merck KGaA, Darmstadt, Germany), respectively.

### 3.2. Plant Material

The twigs of *P.* *nagi* were collected in Ledong County, Hainan Province, China, and identified by Professor Chang-Qiang Ke, Shanghai Institute of Materia Medica. A voucher specimen (No. 20140611) was deposited at the herbarium of Shanghai Institute of Materia Medica, Chinese Academy of Sciences.

### 3.3. Extraction, Isolation, and Characterization

Air-dried twigs of *P.* *nagi* (39.3 kg) were grounded and extracted with 95% EtOH (3 × 35 L) at room temperature (each 72 h). The concentrated extract (1.1 kg) was suspended in water and then partitioned with petroleum ether and EtOAc successively. The EtOAc extract (85 g) was separated into five fractions (Fr.1A‒Fr.1E) by MCI gel CC eluted with EtOH/H_2_O (from 20:80 to 95:5). The 40% ethanol fraction (Fr.1B, 17 g) was subjected to silica gel CC eluted with CH_2_Cl_2_/MeOH (from 80:1 to 10:1) to yield 10 fractions (Fr.1B1–Fr.1B10). Fr.1B5 was purified by CC over Sephadex LH-20 gel eluted with CHCl_3_/MeOH (1:1, *v*/*v*) to obtain **1** (8 mg). Fr.1B10 was subjected to Sephadex LH-20 gel eluted with MeOH to yield Fr.1B10A and Fr.1B10B, which were further purified by preparative HPLC (MeCN/H_2_O, 5%-35%, 0−110 min, 25.0 mL/min) to obtain **4** (6 mg) and **2** (10 mg), respectively. The water fraction was separated into two fractions (Fr.2B and Fr.2C) by macro porous resin AB-8 gel CC with EtOH/H_2_O (40:60 and 70:30). Fr.2C was subjected to silica gel CC eluted with CH_2_Cl_2_/MeOH (from 40:1 to 20:1) to yield two fractions (Fr.2C1 and Fr.2C2). Fr.2C2 was purified by Sephadex LH-20 gel eluted with CHCl_3_/MeOH (1:1, *v*/*v*) to obtain **3** (190 mg).

*1β,16-Dihydroxylambertic acid* (**1**): white powder; [α]${}_{D}^{20}$ +119.8 (*c* 1.0, MeOH); UV (MeOH) λ_max_(log ε), nm: 210 (4.84), 227 (4.62), 265 (3.66); CD (MeOH) λ_max_ (Δε), nm: 211 (4.11), 227 (3.97), 265 (−2.95); IR (KBr, cm^−1^): ν_max_ 3430, 2961, 1695, 1616, 1506, 1418, 1384, 1251; ^1^H- and ^13^C-NMR data, see Table 1; ESI-MS *m*/*z* 349.2 [M + H]^+^ ; HR-ESI-MS *m*/*z* 349.2001 [M + H]^+^ (calcd. for C_20_H_29_O_5_, 349.2010).

*3β,16-Dihydroxylambertic acid* (**2**): white powder; [α]${}_{D}^{20}$ +25.7 (*c* 1.0, MeOH); UV (MeOH) λ_max_(log ε), nm: 210 (4.64), 227 (4.49), 265 (3.31); CD (MeOH) λ_max_ (Δε), nm: 211 (4.14), 227 (3.79), 265 (−2.99); IR (KBr, cm^−1^): ν_max_ 3531, 3385, 2953, 2926, 1687, 1616,1470, 1422, 1383, 1200; ^1^H- and ^13^C-NMR data, see Table 1; ESI-MS *m*/*z* 349.2 [M + H]^+^ ; HR-ESI-MS *m*/*z* 349.2003 [M + H]^+^ (calcd. for C_20_H_29_O_5_, 349.2010).

*ent-2β,15,16,18-Tetrahydroxypimar-8(14)-ene* (**3**): white powder; [α]${}_{D}^{20}$ +8.8 (*c* 1.0, MeOH); IR (KBr, cm^−^^1^): ν_max_ 3424, 2932, 2872, 1769, 1720, 1646, 1459, 1383; ^1^H- and ^13^C-NMR data, see Table 2; ESI-MS *m*/*z* 383.2 [M + HCOO]^−^; HR-ESI-MS *m*/*z* 383.2438 [M + HCOO]^–^ (calcd. for C_21_H_35_O_6_, 383.2439).

*ent-15-oxo-2β,16,18-Trihydroxypimar-8(14)-ene* (**4**): white powder; [α]${}_{D}^{20}$ +3.8 (*c* 1.0, MeOH); IR (KBr, cm^–1^): ν_max_ 3426, 2929, 2870, 1710, 1618, 1459, 1385; ^1^H- and ^13^C-NMR data, see Table 2; ESI-MS *m*/*z* 337.2 [M + H]^+^; HR-ESI-MS *m*/*z* 337.2374 [M + H]^+^(calcd. for C_20_H_33_O_4_, 337.2373).

### 3.4. Reagents

The compounds were dissolved in dimethyl sulfoxide (DMSO, Sigma-Aldrich, St. Louis, MO, USA) as a stock solution and stored at −20 °C. Dulbecco′s modified Eagle′s medium (DMEM), RPMI 1640 medium, 0.25% trypsin-EDTA, fetal bovine serum (FBS), Penicillin-Streptomycin (10,000 units/mL of penicillin and 10,000 μg/mL of streptomycin), and phosphate-buffered saline (PBS) were purchased from Gibco (Carlsbad, CA, USA). Paclitaxel, 3-(4,5-dimethyl-2-thiazolyl)-2,5-diphenyltetrazolium bromide (MTT), indomethacin, and LPS were purchased from Sigma-Aldrich (St. Louis, MO, USA).

### 3.5. Cell Culture

The human cervical cancer Hela cells were acquired from American Type Culture Collection (Rockville, MD, USA) and cultured in DMEM medium supplemented with 10% (*v*/*v*) FBS and 1% (*v*/*v*) Penicillin-Streptomycin. The human non-small cell lung cancer A549 cells were obtained from American Type Culture Collection (Rockville, MD, USA) and cultured in RPMI 1640 medium containing 10% (*v*/*v*) FBS and 1% (*v*/*v*) Penicillin-Streptomycin. The human breast cancer MCF-7 cells were acquired from KeyGEN Biotech (Nanjing, Jiangsu, China) and cultured in DMEM medium supplemented with 10% (*v/v*) FBS and 1% (*v*/*v*) Penicillin-Streptomycin. The murine macrophage RAW264.7 cells were obtained from American Type Culture Collection (Rockville, MD, USA) and maintained in DMEM supplemented with 10% FBS. Cells were grown in a standard humidified incubator with 5% CO_2_ at 37 °C.

### 3.6. MTT Assay

Viability of the cells after treatment with the compounds was determined by MTT assay as described previously [19]. Exponentially growing Hela, A549, MCF-7, and RAW264.7 cells were seeded into 96-well plates. Upon approximately 70%–80%, the cells were treated with series concentrations of different compounds (48 h for Hela, A549 and MCF-7 cells; 24 h for RAW264.7 cells). Paclitaxel was used as the positive control, and DMSO was used as the vehicle control. Then, a 1-mg/mL MTT solution was added to each well and the 96-well plates were further incubated for 4 h at 37 °C. A 100-μL portion of DMSO was added to each well to dissolve the formazan crystals. Absorbance at 570 nm was measured by a microplate reader (Perkin Elmer, 1420 Multilabel Counter Victor 3, Wellesley, MA, USA).

### 3.7. NO Production Assay

RAW264.7 cells were seeded into 24-well plates (1 × 10^5^ cells/well). After 24 h, the cells were treated with different concentrations of compounds or vehicle (DMSO) for 1 h. Then, the cells were stimulated with 1 μg/mL LPS and further incubated for 18 h. Indomethacin was used as the positive control. To measure the NO content, the culture supernatant was collected and assayed using Griess reagent, as described previously [20].

### 3.8. Statistical Analysis

Data were expressed as mean values and standard deviation. All the experiments were repeated under the same conditions at least three times (*n* = 9). Statistical significances were analyzed by one-way analysis of variance using SPSS 17 software (Statistical Package for the Social Sciences, SPSS Inc., Chicago, IL, USA). *p* < 0.05 was considered as the significant difference.

## 4. Conclusions

In conclusion, four new compounds were isolated from the twigs of *P. nagi*, including two abietane-type diterpenoids, 1β,16-dihydroxylambertic acid, and 3β,16-dihydroxylambertic acid, and two *ent*-pimarane-type diterpenoids, *ent*-2*β*,15,16,18-tetrahydroxypimar-8(14)-ene and *ent*-15-oxo-2*β*,16,18-trihydroxypimar-8(14)-ene. *ent*-Pimarane-type diterpenoids have rarely been reported in plants of the family Podocarpaceae. To the best of our knowledge, it is the first report of *ent*-pimarane-type diterpenoids from plants of the genus *Podocarpus*. The discovery of *ent*-pimarane type diterpenoids enriches the chemical composition diversity of the genus *Podocarpus*. Additionally, two *ent*-pimarane type diterpenoids exhibited significant cytotoxic activity against three human cancer cell lines, and compounds **2** and **4** suppressed LPS-induced NO production on RAW264.7 cells, which might be developed as anti-cancer and anti-inflammation agents.

## Figures and Tables

**Table 1:** ^1^H- and ^13^C-NMR data (δ in ppm) for compounds **1** and **2**.

NO.	1 (CD_3_OD) ^a^		2 (CD_3_OD) ^b^	
	δ_H_ (*J* in Hz)	δ_C_	δ_H_ (*J* in Hz)	δ_C_
1	3.52, dd (11.7, 4.9)	79.1, CH	α: 1.74, m; β: 1.89, m	34.2, CH_2_
2	α: 1.69, dt (12.1, 4.9); β: 2.07, m	31.5, CH_2_	α: 1.71, m; β: 2.30, m	28.2, CH_2_
3	α: 1.18, m; β: 2.18, dd (13.2, 3.5)	37.0, CH_2_	4.05, dd (4.5, 2.2)	71.3, CH
4		44.3, C		48.9, C
5	1.39, dd (11.2, 1.9)	53.9, CH	1.91, m	46.4, CH
6	α: 2.01, m; β: 2.08, m	22.4, CH_2_	α: 2.00, m; β: 2.01, m	22.3, CH_2_
7	2.65, dd (6.9, 3.5), 2H	33.5, CH_2_	α: 2.66, d (6.8); β: 2.75, m	32.4, CH_2_
8		136.1, C		127.3, C
9		139.7, C		148.1, C
10		45.0, C		39.2, C
11	8.06, s	130.0, CH	6.65, s	112.9, CH
12		153.7, C		154.0, C
13		129.0, C		129.3, C
14	6.36, s	115.5, CH	6.71, s	128.9, CH
15	3.13, dd (7.2, 5.8)	37.6, CH	3.15, q (6.8)	36.9, CH
16	3.48, dd (10.2, 7.4) 3.69, dd (10.2, 5.7)	68.4, CH_2_	3.49, dd (10.5, 7.4) 3.69, dd (10.5, 5.8)	68.2, CH_2_
17	1.22, d (6.8)	17.3, CH_3_	1.21, d (7.0)	17.1, CH_3_
18	1.24, s	29.1, CH_3_	1.31, s	24.7, CH_3_
19		181.2, C		181.6, C
20	1.13, s	18.2, CH_3_	1.09, s	23.3, CH_3_

^a^ Measured at 500 MHz for ^1^H-NMR and 125 MHz for ^13^C-NMR. ^b^ Measured at 600 MHz for ^1^H-NMR and 150 MHz for ^13^C-NMR.

**Table 2:** ^1^H- and ^13^C-NMR data (500 and 125 MHz, resp. δ in ppm) of compounds **3** and **4**.

NO.	3 (CD_3_OD)		4 (CD_3_OD)	
	δ_H_ (*J* in Hz)	δ_C_	δ_H_ (*J* in Hz)	δ_C_
1	α: 1.01, m; β: 2.02, m	48.8, CH_2_	α: 0.98, m; β: 2.00, m	48.8, CH_2_
2	3.85, m	65.7, CH	3.82, m	65.6, CH
3	α: 1.46, m; β: 1.60, m	45.3, CH_2_	α: 1.44, m; β: 1.59, m	45.3, CH_2_
4		40.3, C ^a^		40.3, C
5	1.43, m	47.8, CH	1.46, m	47.7, CH
6	α: 1.27, t (12.6, 5.0); β: 1.51, m	23.1, CH_2_	α: 1.29, dd (13.0, 4.5); β: 1.54, m	23.0, CH_2_
7	α: 2.16, m; β: 2.28, m	36.5, CH_2_	α: 2.15, dd (12.7, 6.2); β: 2.29, dd (12.7, 4.0)	36.5, CH_2_
8		138.8, C		141.4, C
9	1.87, d (8.6)	52.3, CH	1.89, d (7.8)	51.5, CH
10		40.4, C ^a^		40.7, C
11	α: 1.55, m; β: 1.69, m	19.7, CH_2_	α: 1.57, m; β: 1.65, dd (6.9, 2.7)	19.8, CH_2_
12	α: 1.36, d (14.7); β: 1.52, m	31.2, CH_2_	α: 1.52, m; β: 1.70, dd (12.2, 2.7)	32.3, CH_2_
13		39.1, C		48.1, C
14	5.37, s	129.3, CH	5.49, s	125.0, CH
15	3.34, m	80.8, CH		215.3, C
16	3.38, m; 3.74, dd (11.1, 2.4)	63.9, CH_2_	4.36, d (18.7); 4.32, d (18.7)	65.6, CH_2_
17	0.99, s	23.1, CH_3_	1.17, s	23.9, CH_3_
18	0.85, s	19.3, CH_3_	0.81, s	19.3, CH_3_
19	3.05, d (11.0); 3.36, m	71.7, CH_2_	3.01, d (11.1); 3.38, m	71.6, CH_2_
20	0.90, s	16.9, CH_3_	0.88, s	17.2, CH_3_

^a^ These data may be interchanged.

## Supplementary Materials

The following are available online at www.mdpi.com/1420-3049/21/10/1282/s1. ^1^H-, ^13^C-NMR, HMBC, HSQC, and NOESY spectra of compounds **1**–**4** are available.
