# Supplementary material for: Novel Diterpenoids from the Twigs of Podocarpus nagi
Source: Molecules. 2016 Sep 24;21(10):1282. doi: 10.3390/molecules21101282 (PMC6274396; doi:10.3390/molecules21101282)
Supplement: Supplementary file 1 [file molecules-21-01282-s001.pdf]

Supplementary Materials: Novel Diterpenoids from the Twigs of *Podocarpus nagi*

Yuan-Dong Zheng, Xing-Chen Guan, Dan Li, An-Qi Wang, Chang-Qiang Ke, Chun-Ping Tang, Li-Gen Lin, Yang Ye, Zheng-Liang Wang and Sheng Yao

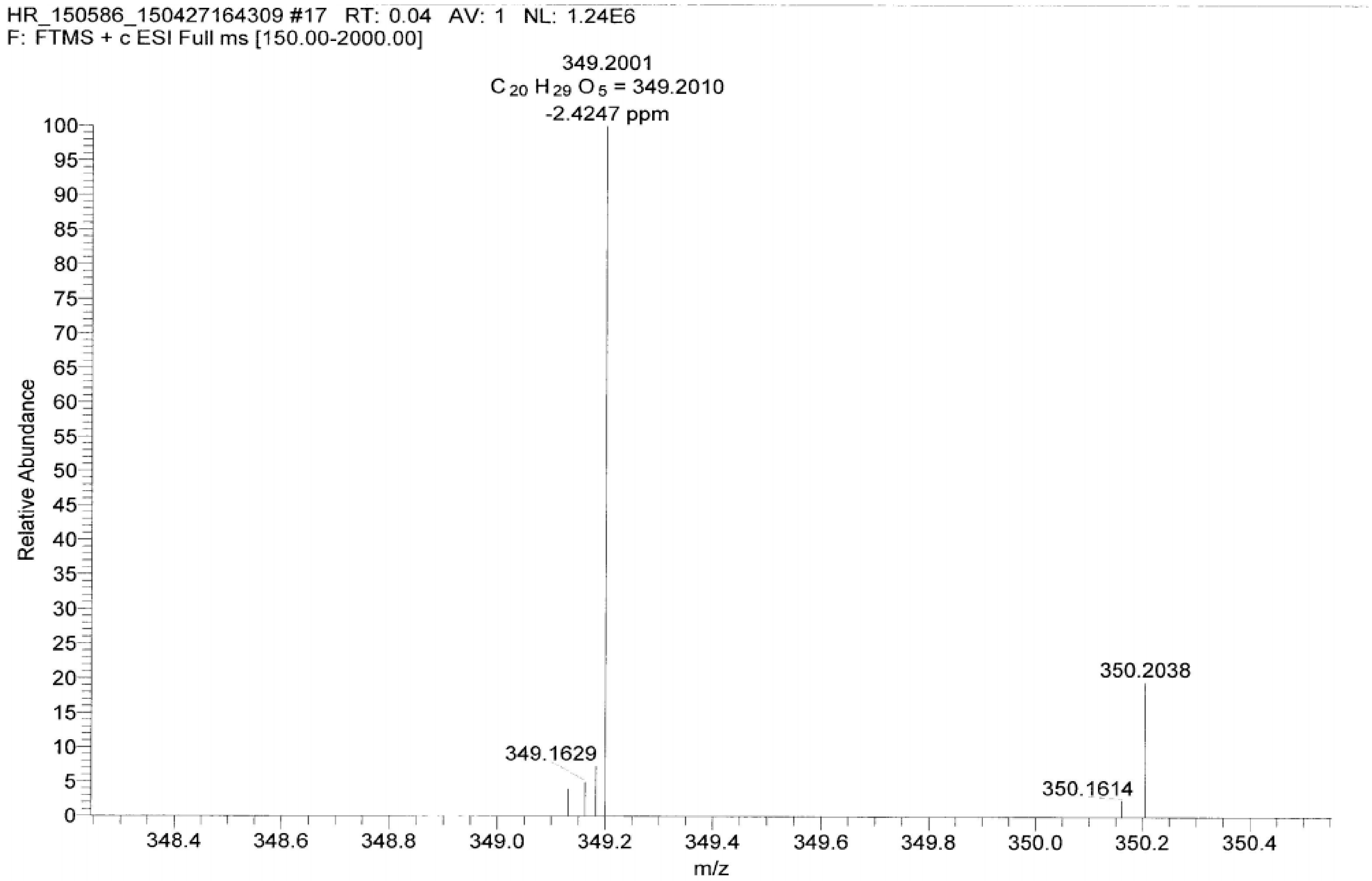

Figure S1. HR-ESI mass spectrum of compound **1**.

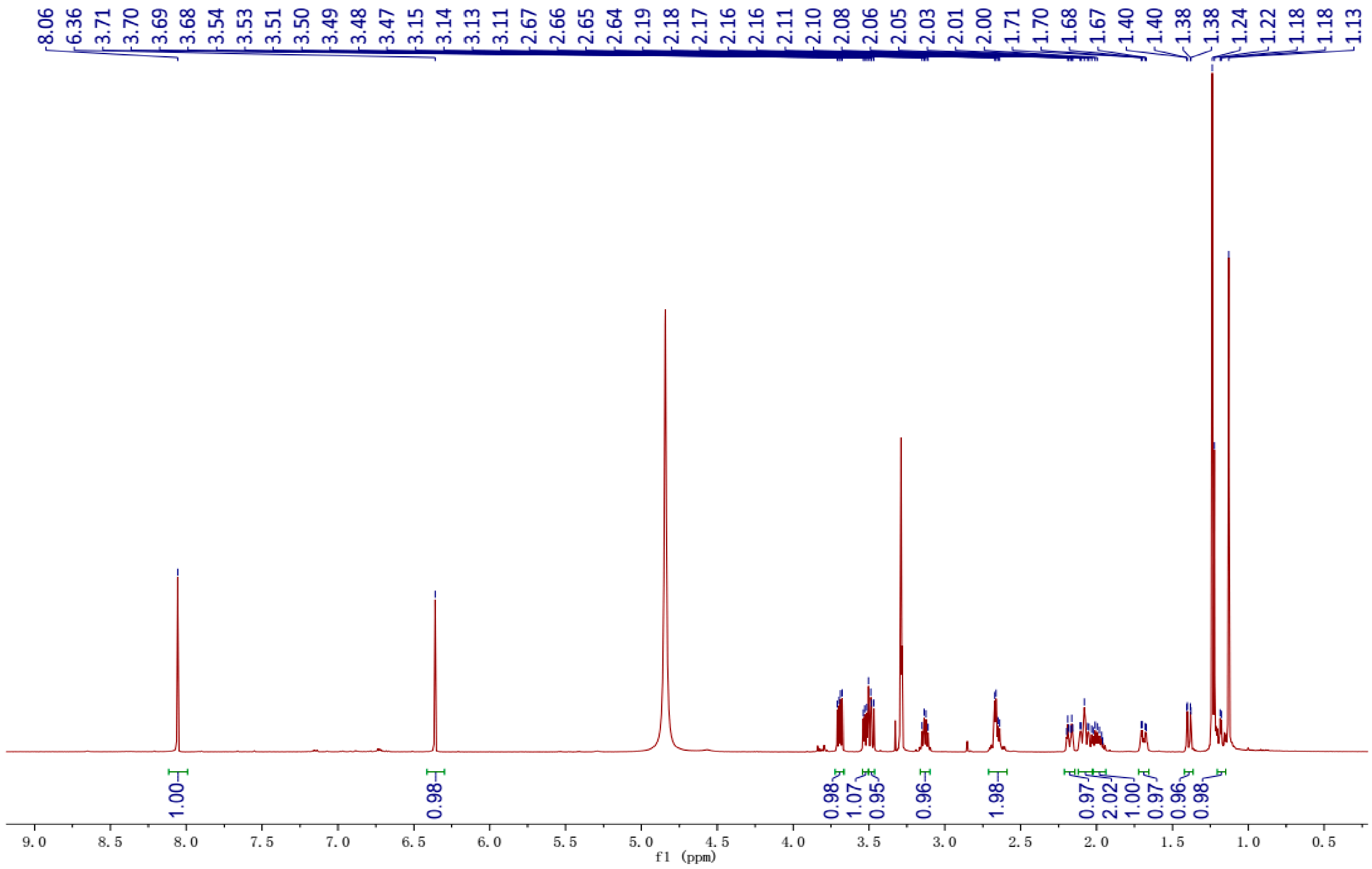

Figure S2. ^{1}H-NMR spectrum of compound **1** in CD_3OD (500 MHz).

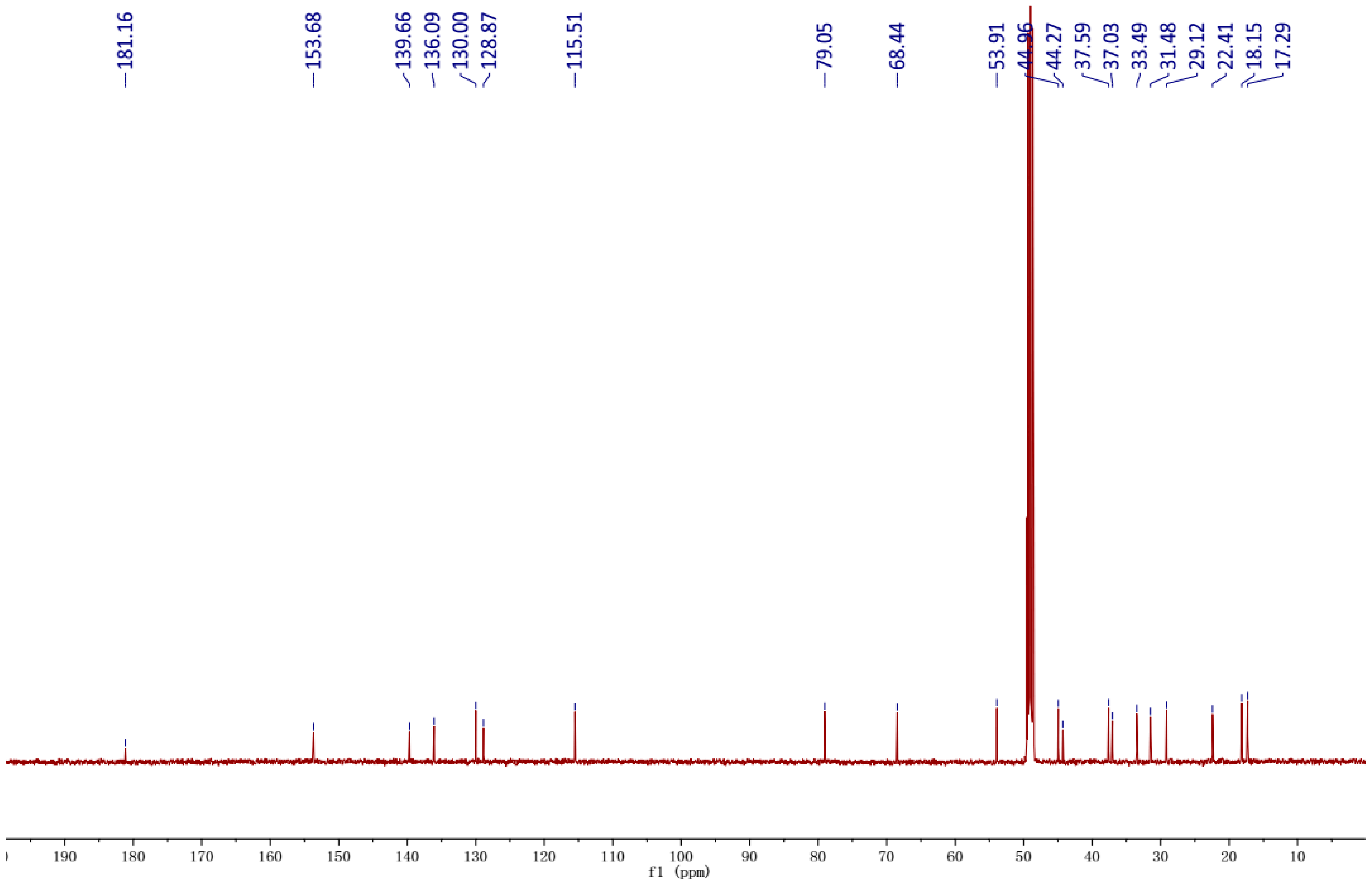

Figure S3. ^{13}C-NMR spectrum of compound **1** in CD_3OD (125 MHz).

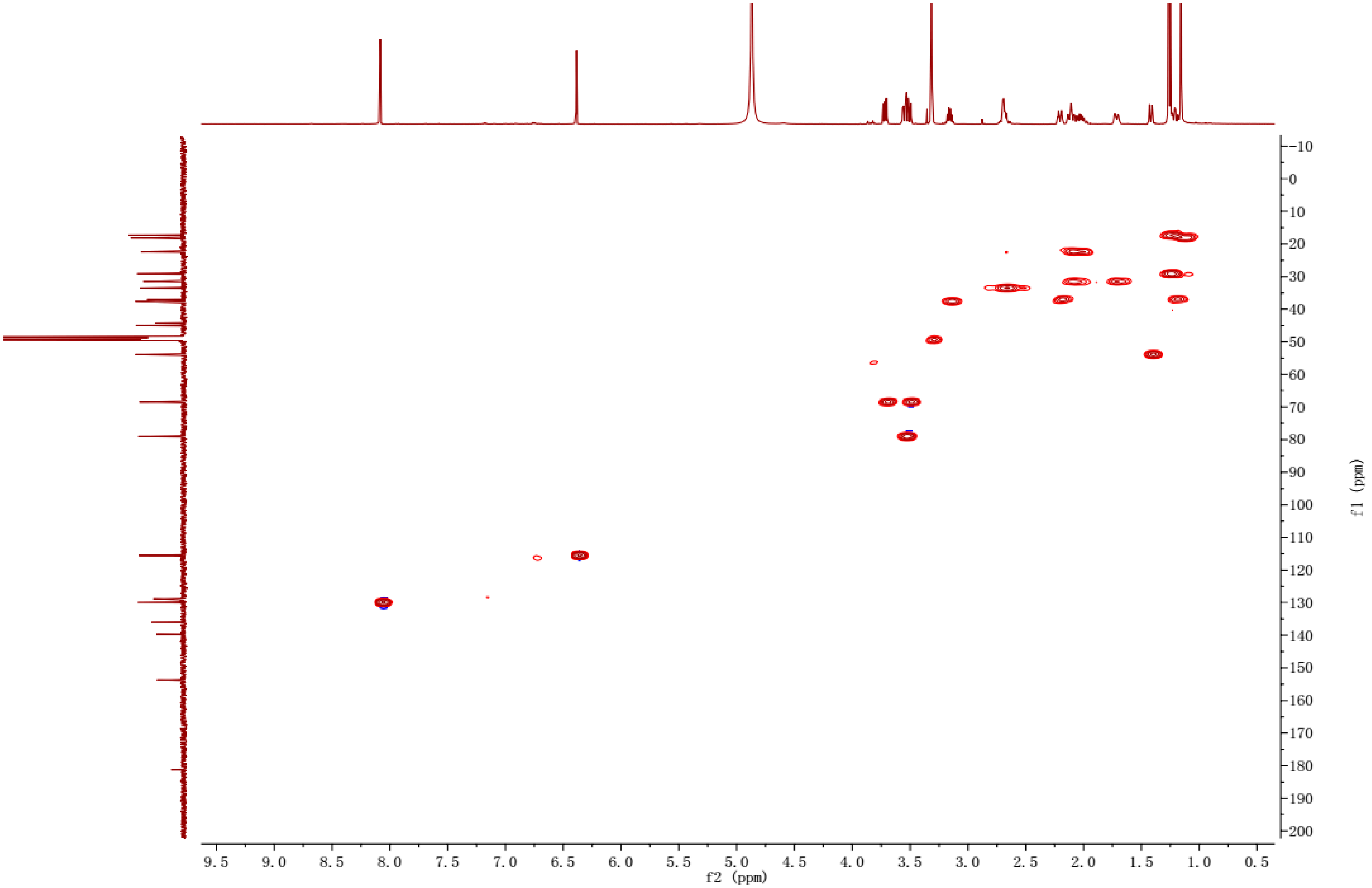

Figure S4. HSQC spectrum of compound **1** in CD_3OD.

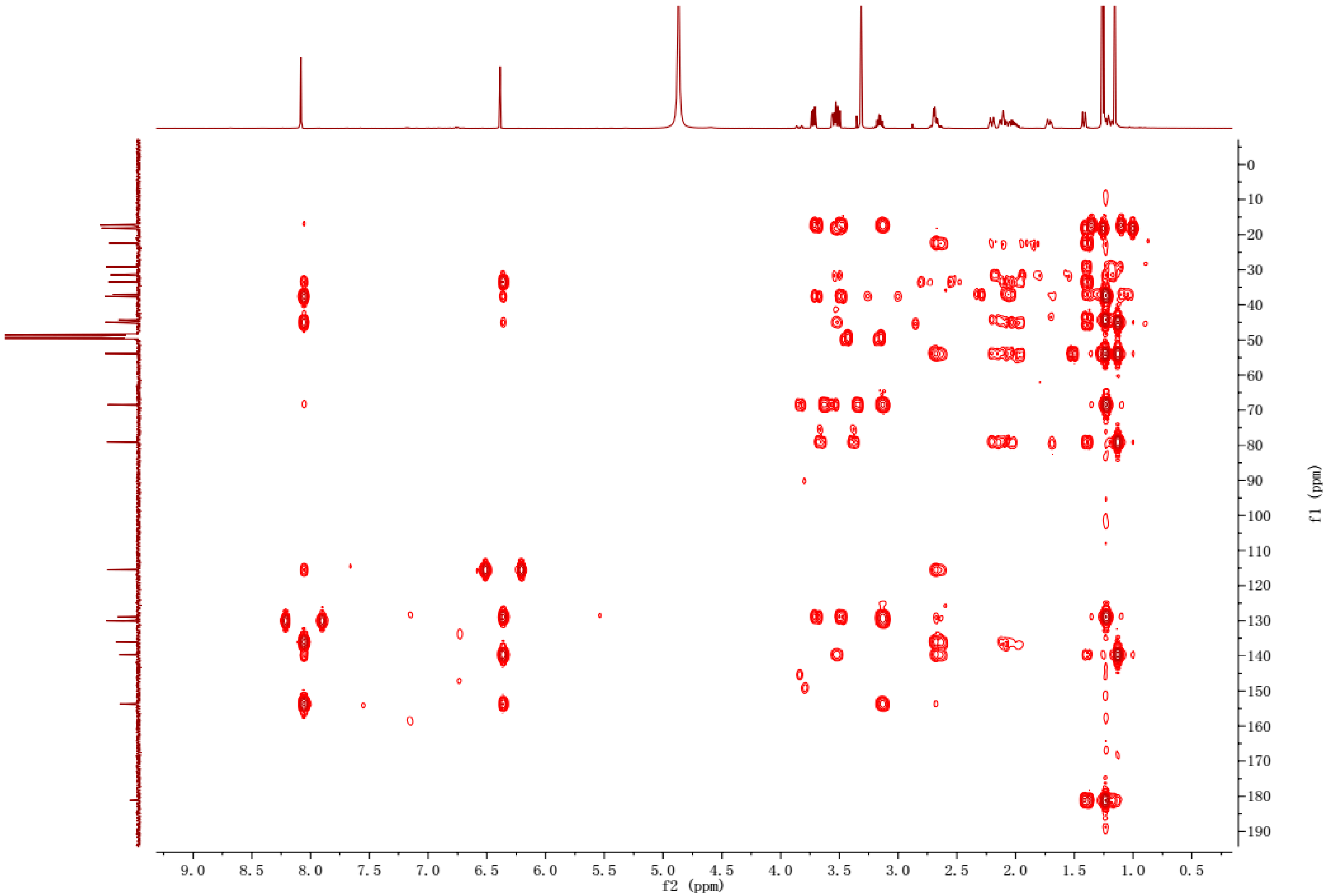

Figure S5. HMBC spectrum of compound **1** in CD_3OD.

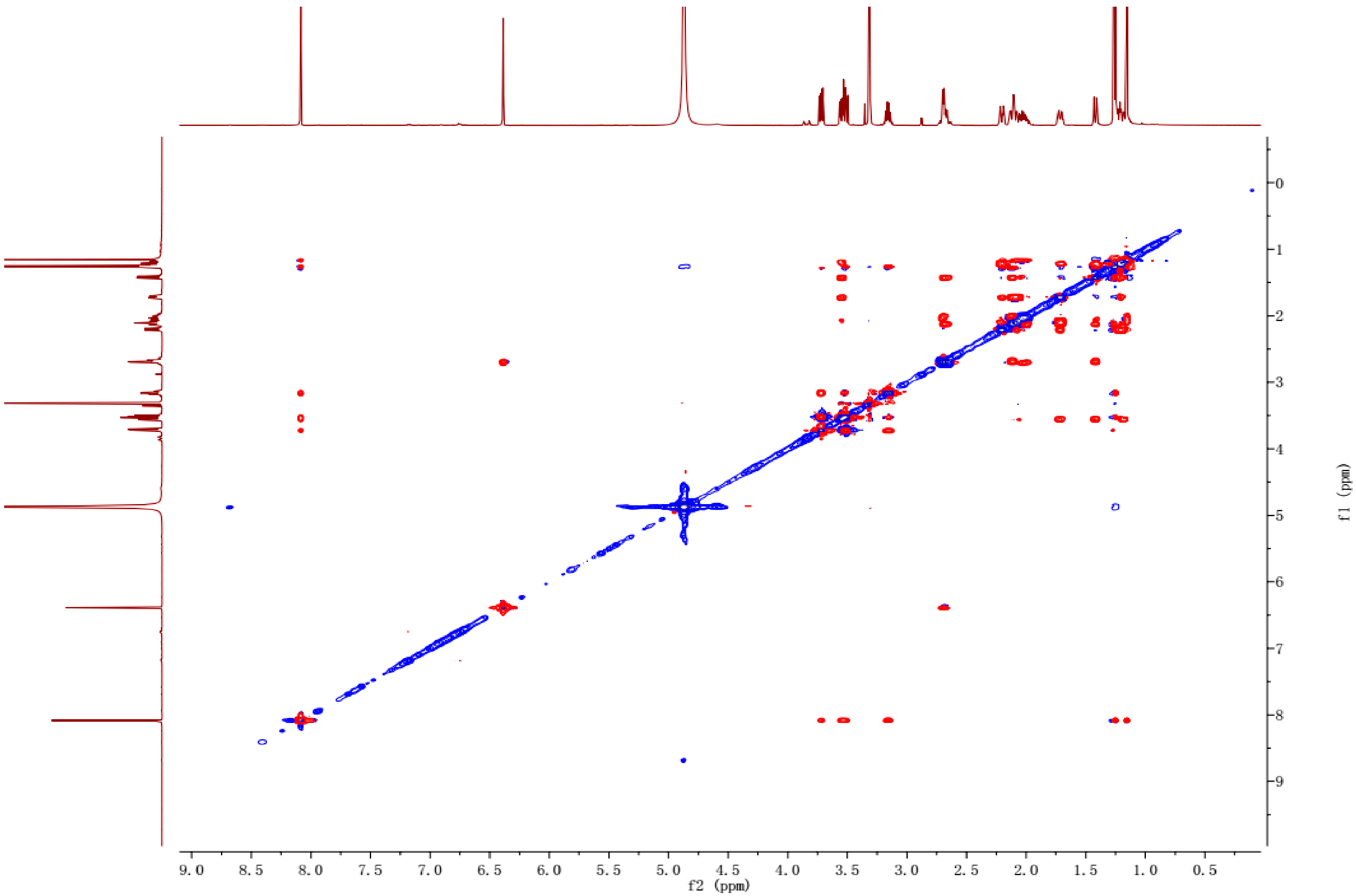

Figure S6. NOESY spectrum of compound **1** in CD_3OD.

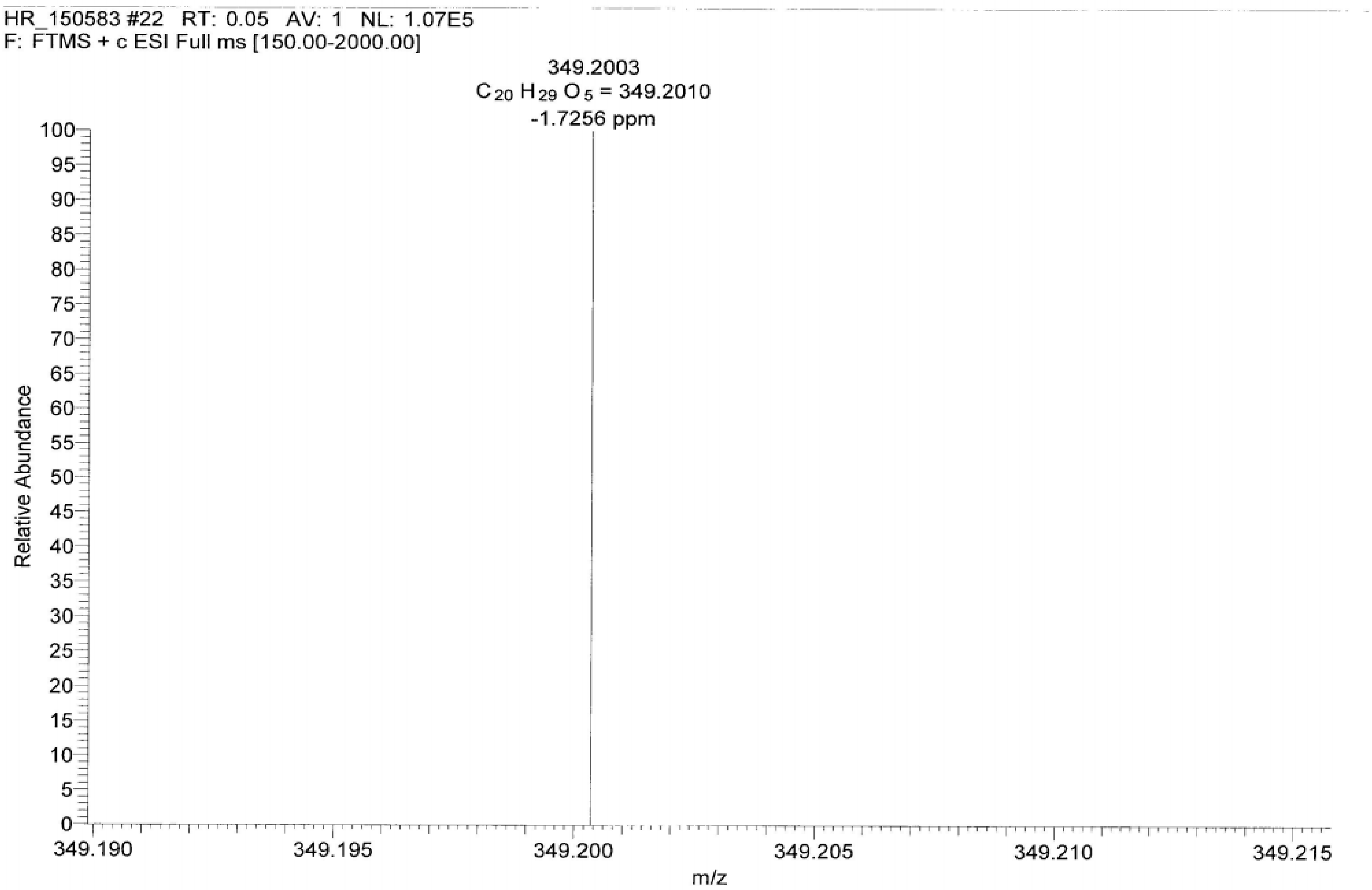

Figure S7. HR-ESI mass spectrum of compound **2**.

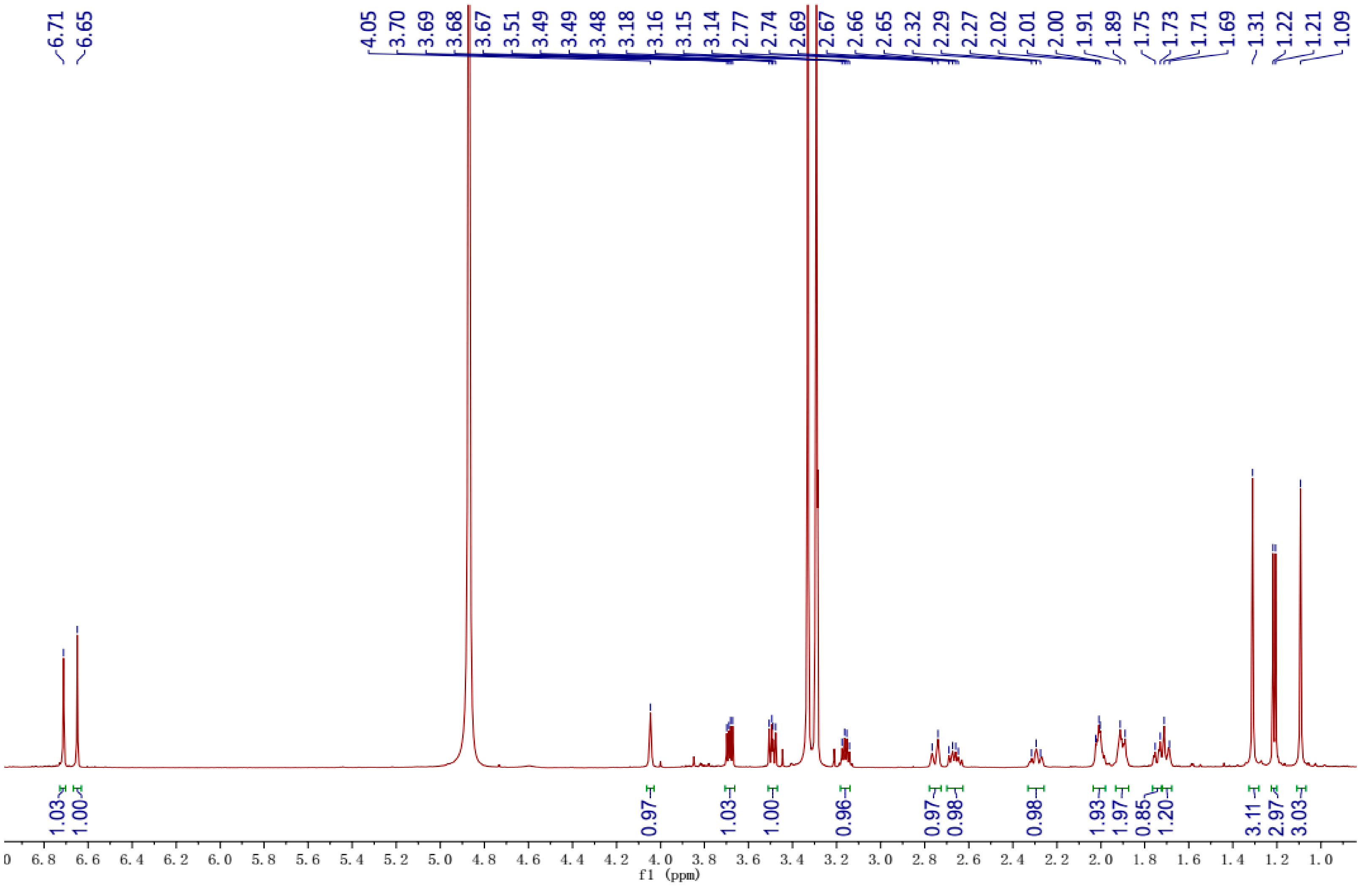

Figure S8. ^{1}H-NMR spectrum of compound **2** in CD_3OD (600 MHz).

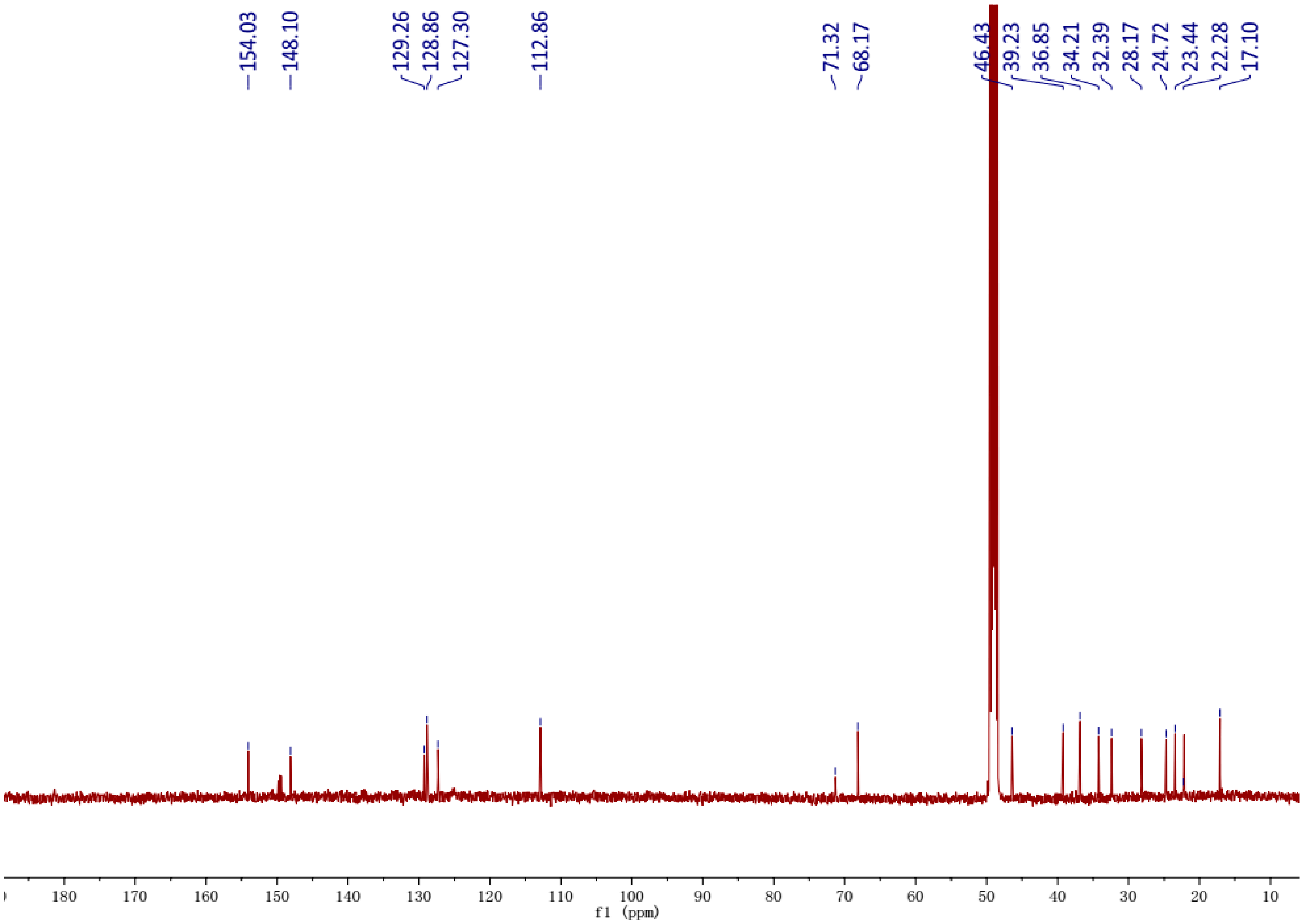

Figure S9. ^{13}C-NMR spectrum of compound **2** in CD_3OD (150 MHz).

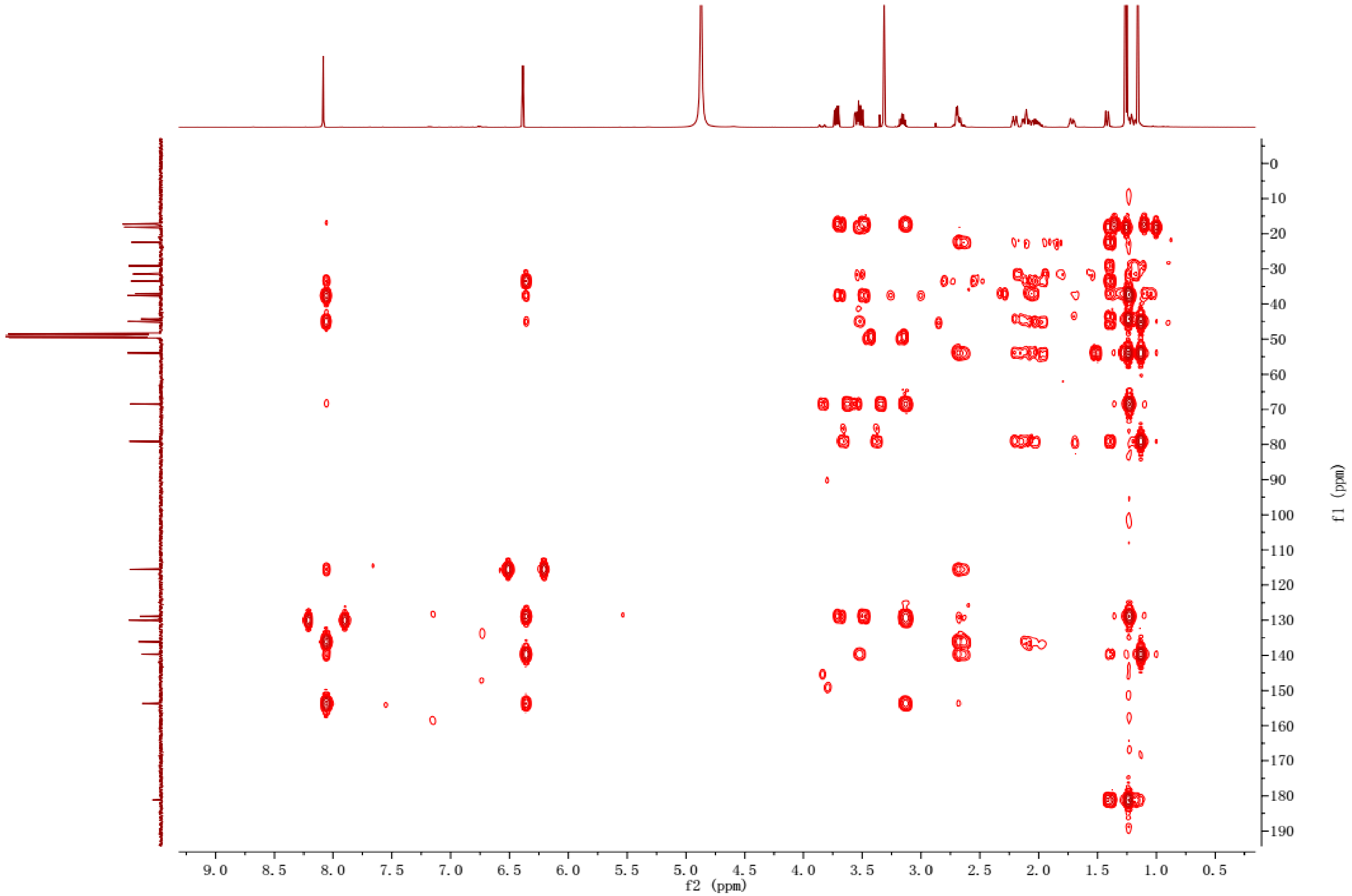

Figure S10. HSQC spectrum of compound **2** in CD_3OD.

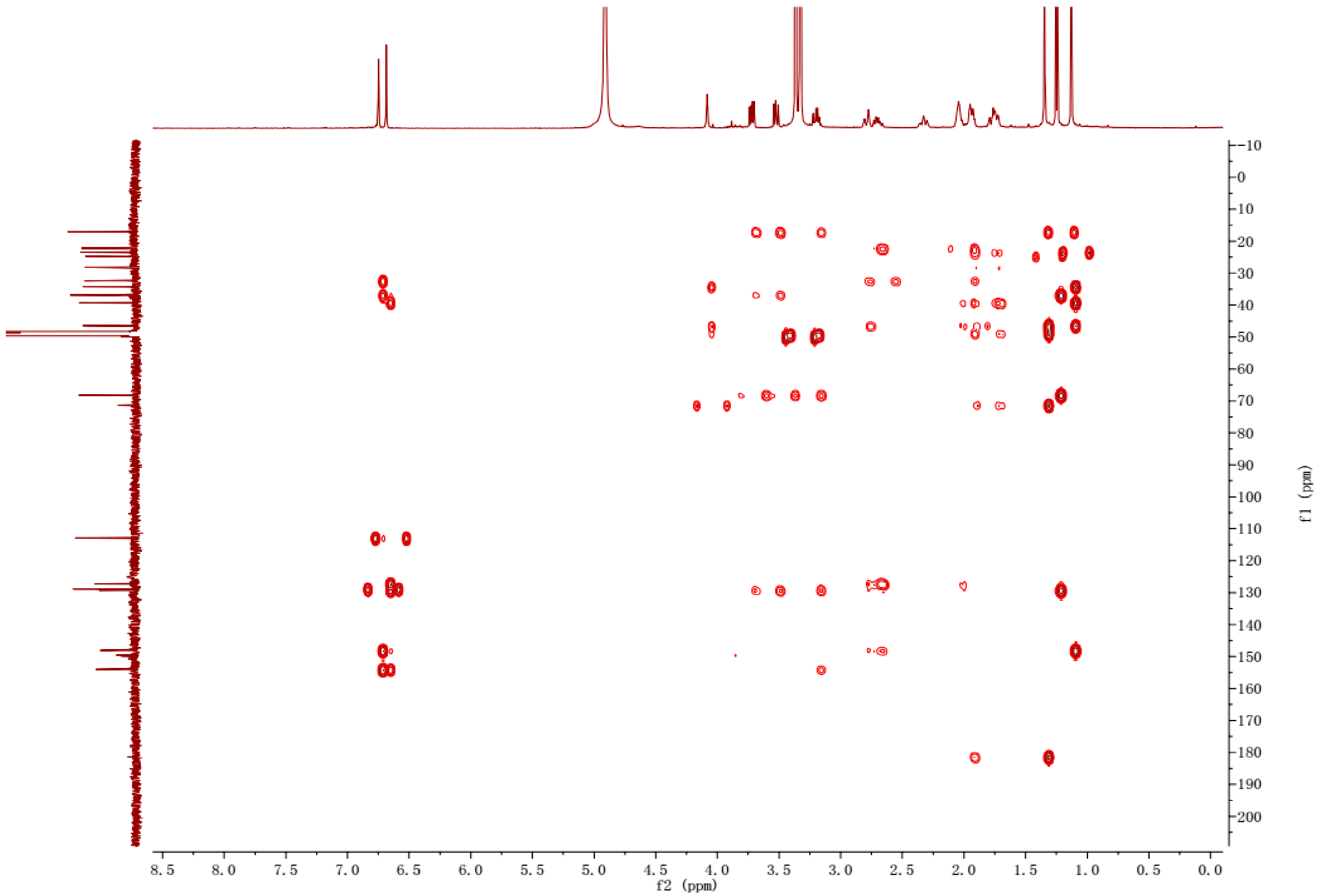

Figure S11. HMBC spectrum of compound **2** in CD_3OD.

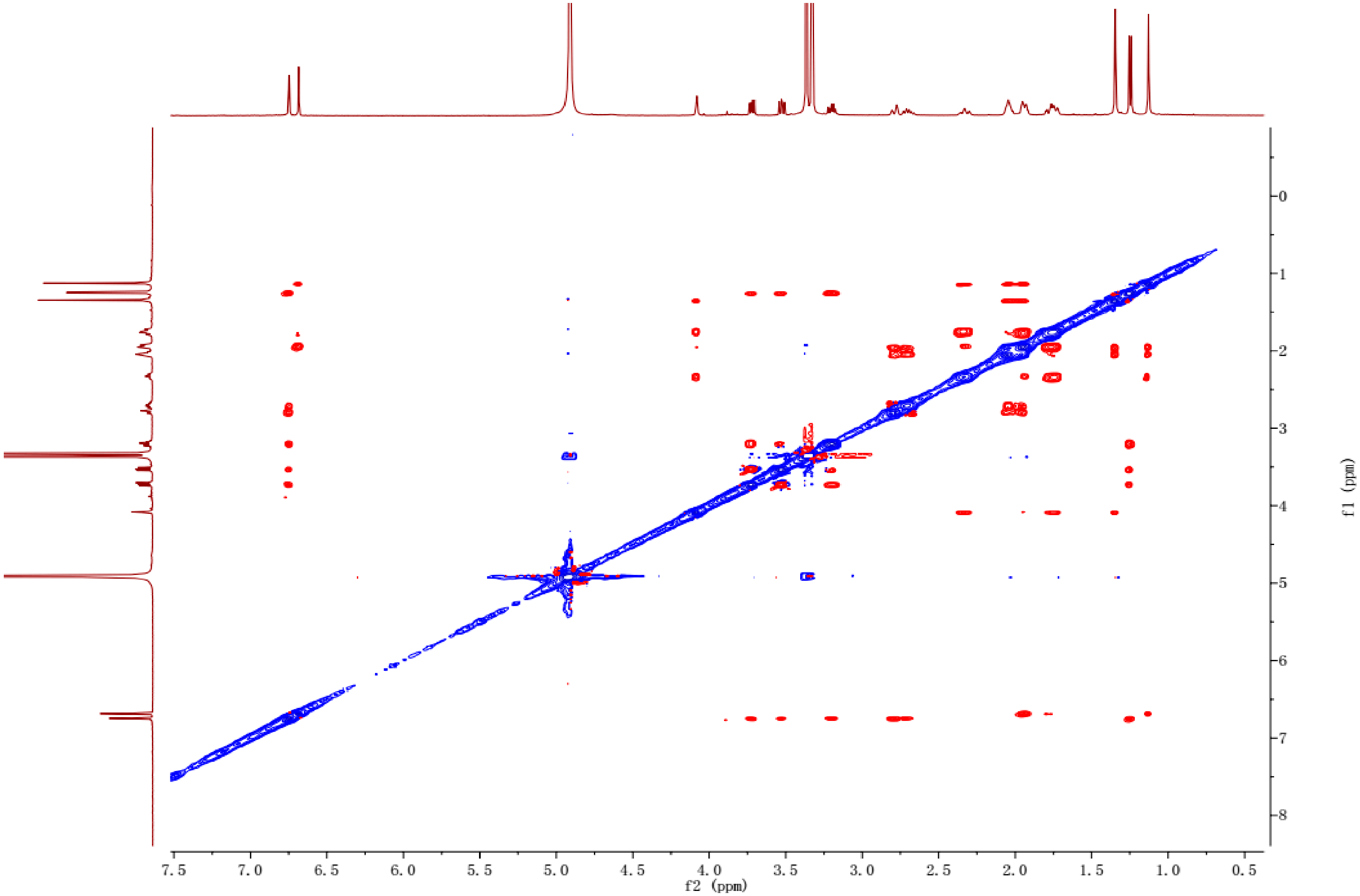

Figure S12. NOESY spectrum of compound **2** in CD_3OD.

Qualitative Analysis Report

**Data Filename**	20160809_ESIH_N_YY_ZYD_161290.d	**Sample Name**	PN-2C2A
**Sample Type**	Sample	**Position**	P1-A2
**Instrument Name**	Agilent G6520 Q-TOF	**Acq Method**	20160324_MS_ESIH_NEG_1min.m
**Acquired Time**	8/9/2016 2:39:36 PM	**IRM Calibration Status**	Success
**DA Method**	small molecular data analysis method.m	**Comment**	ESIH

User Spectra

Spectrum Source	Fragmentor Voltage	Collision Energy	Ionization Mode
	175	0	ESI

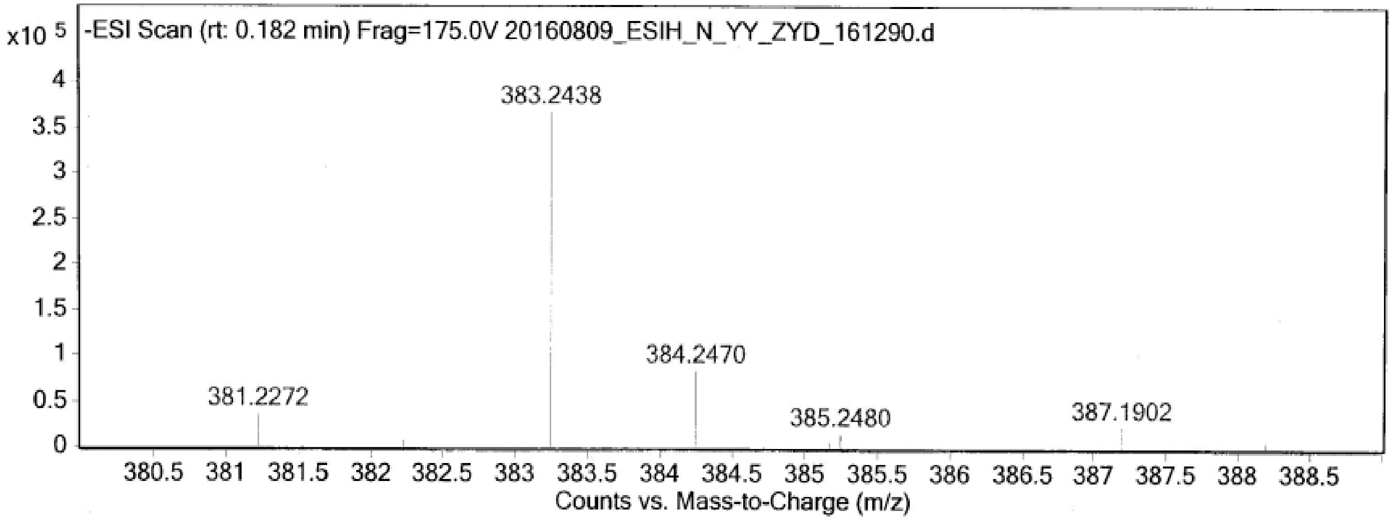

Formula Calculator Results

m/z	Calc m/z	Diff (mDa)	Diff (ppm)	Ion Formula	Ion
383.2438	383.2439	0.11	0.28	C21 H35 O6	(M+COOH)-

--- End Of Report ---

Figure S13. HR-ESI mass spectrum of compound **3**.

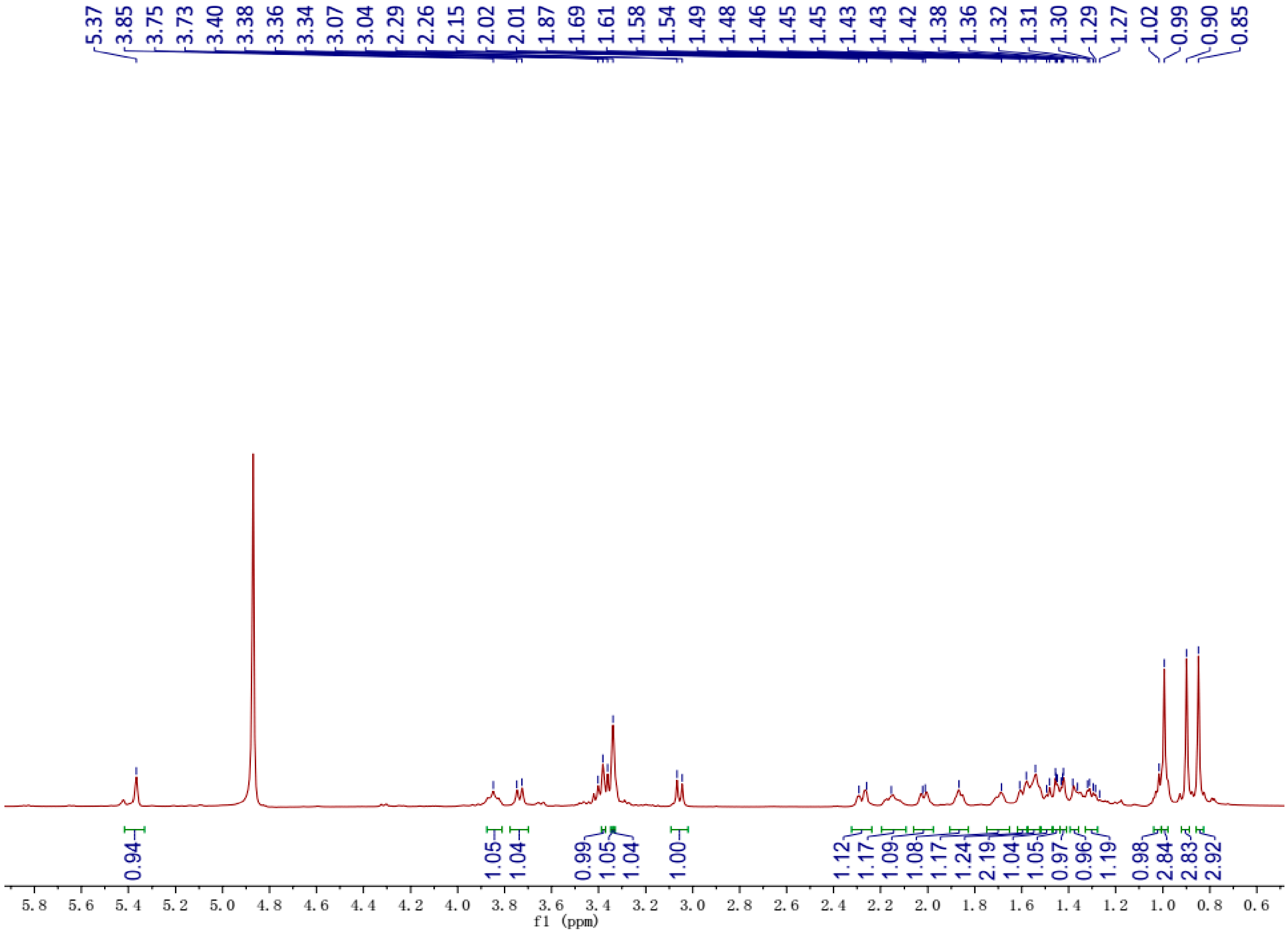

Figure S14. ^{1}H-NMR spectrum of compound **3** in CD_3OD (500 MHz).

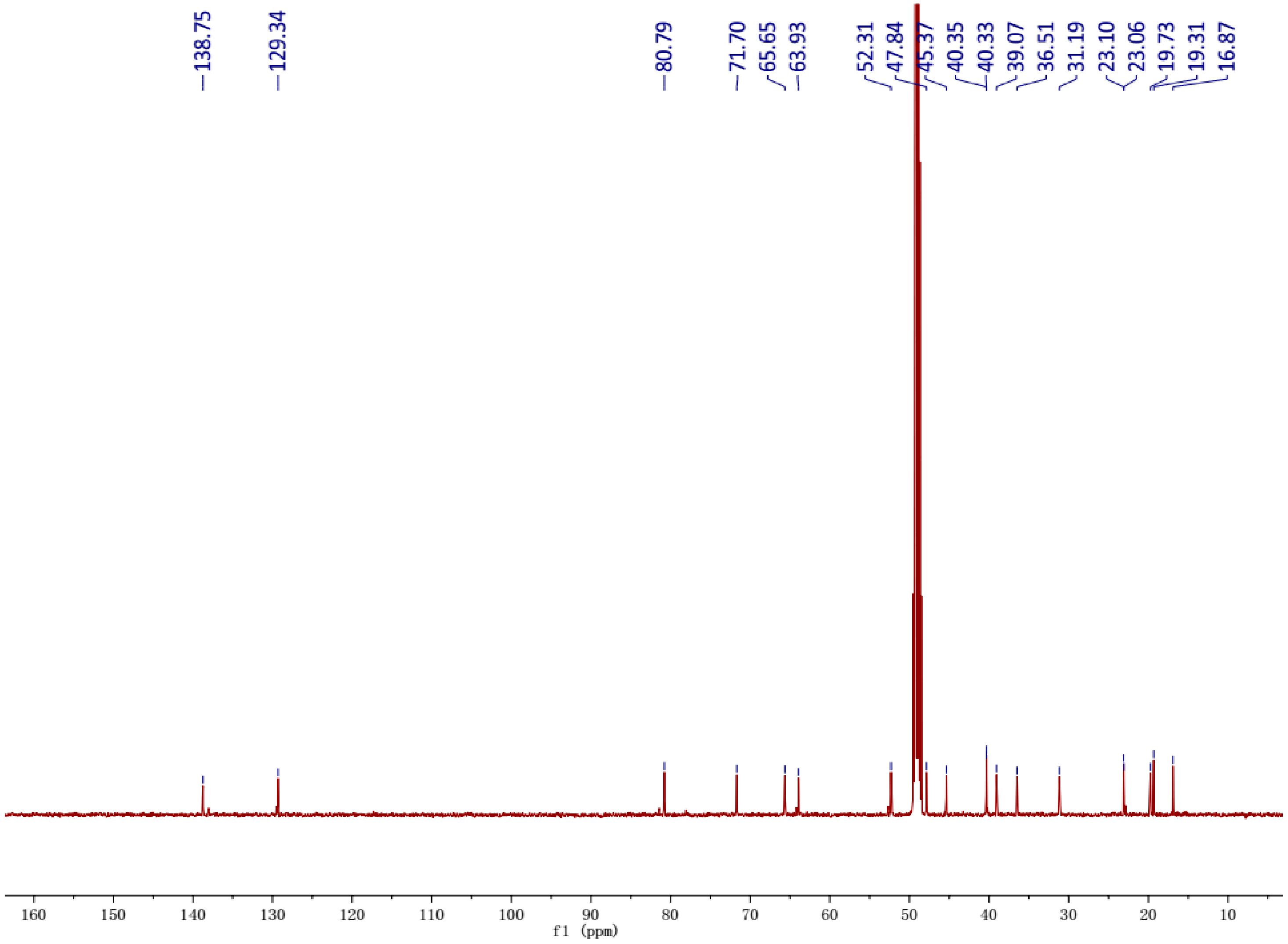

Figure S15. ^{13}C-NMR spectrum of compound **3** in CD_3OD (125 MHz).

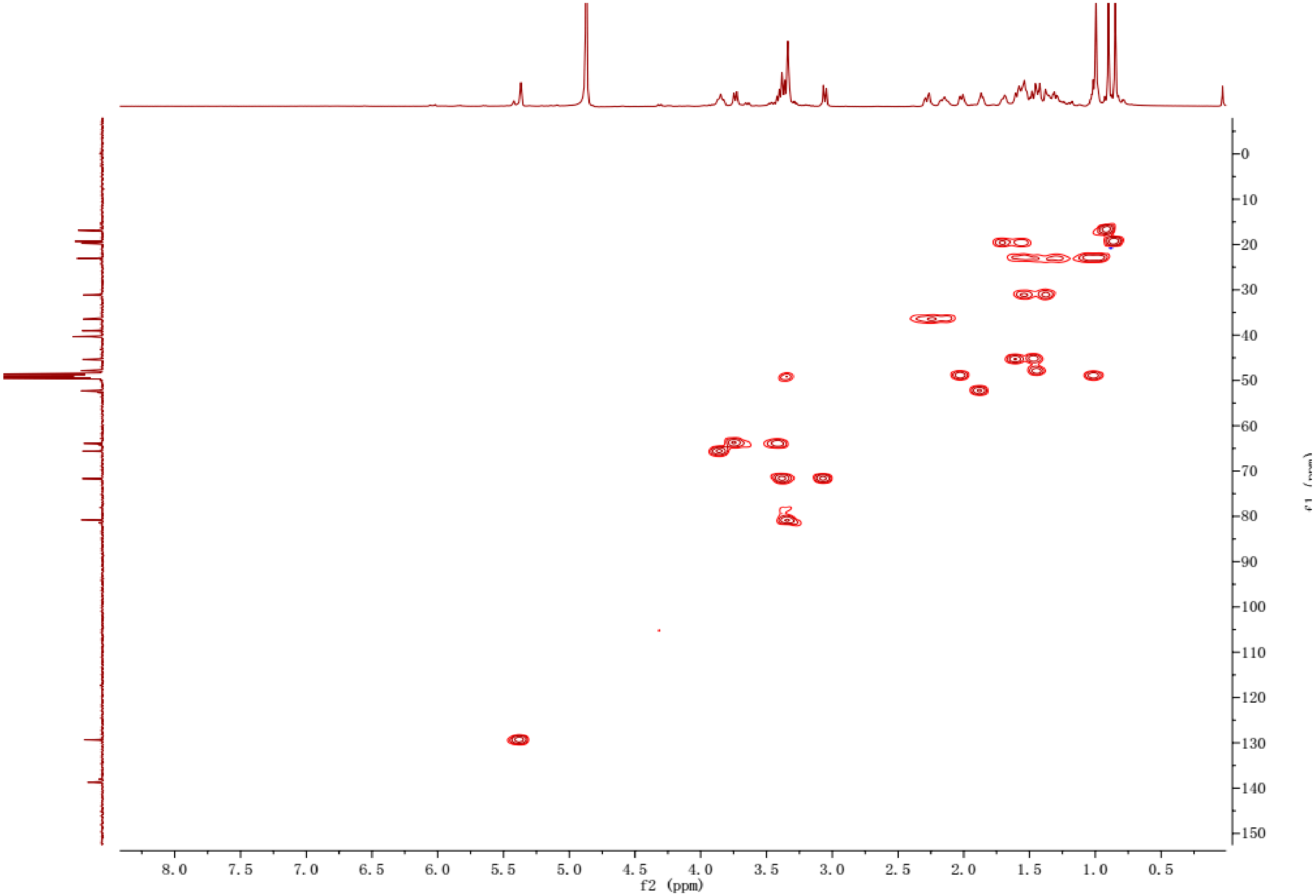

Figure S16. HSQC spectrum of compound **3** in CD_3OD.

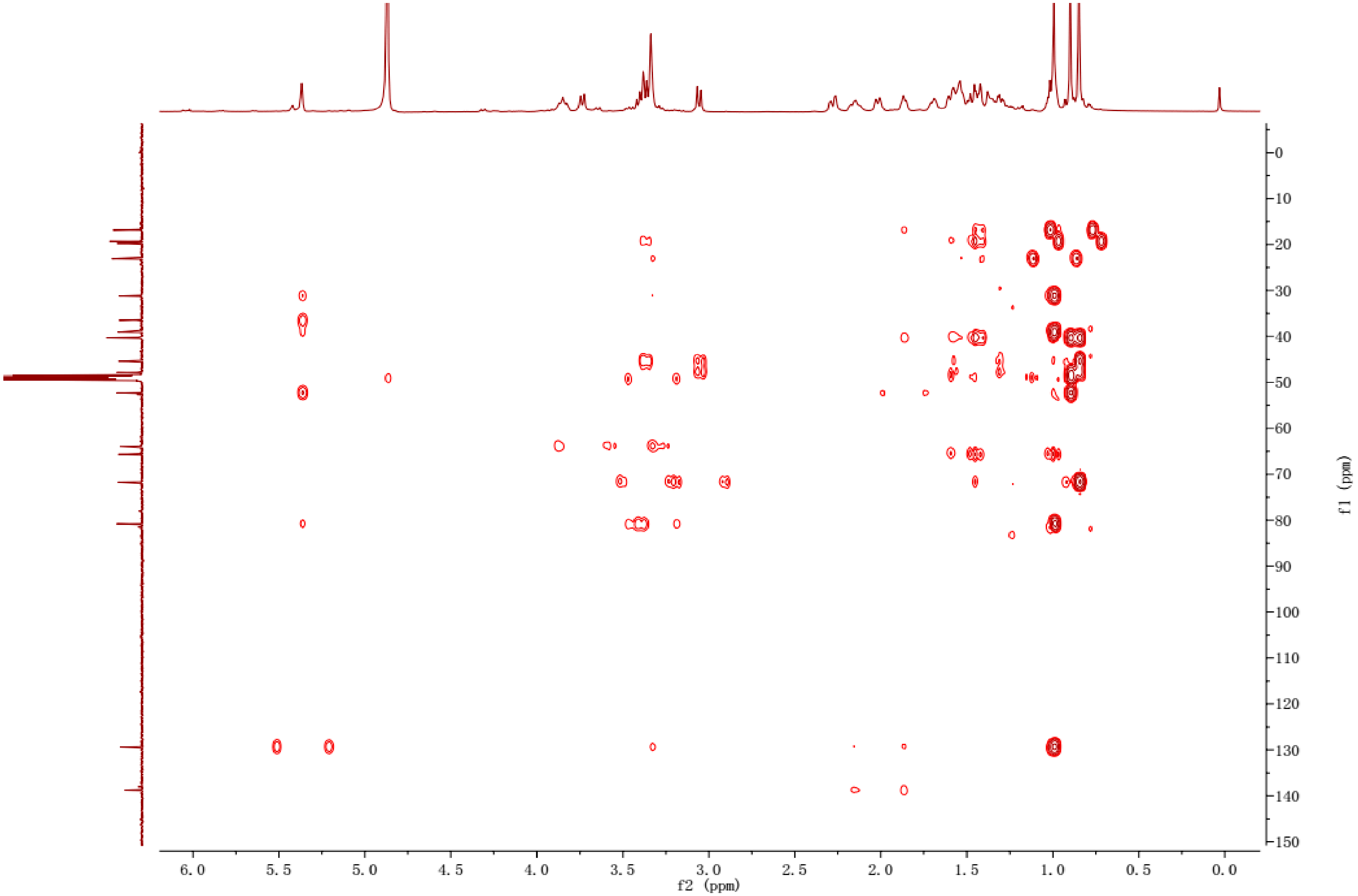

Figure S17. HMBC spectrum of compound **3** in CD_3OD.

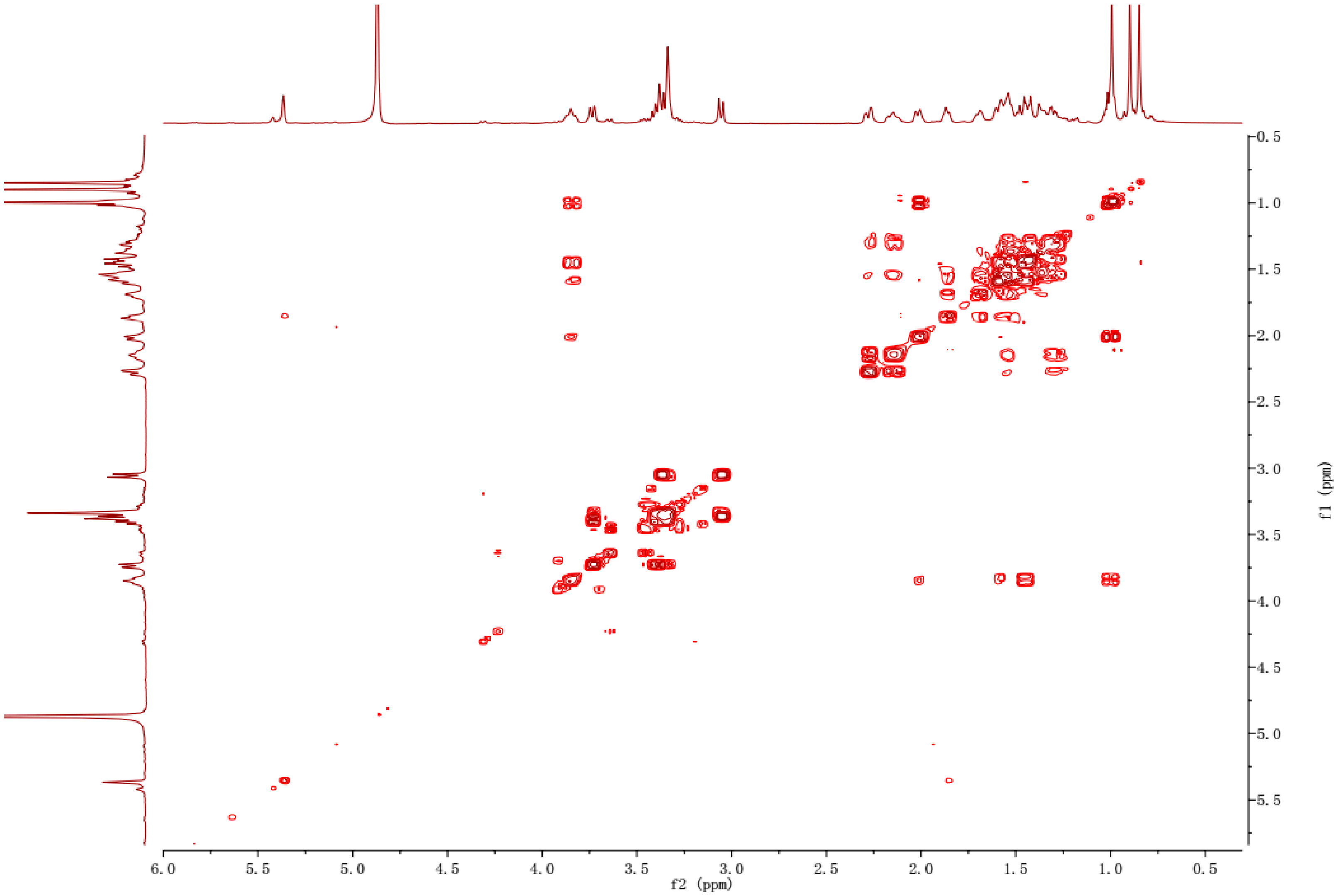

Figure S18. ^{1}H-^{1}H COSY spectrum of compound **3** in CD_3OD.

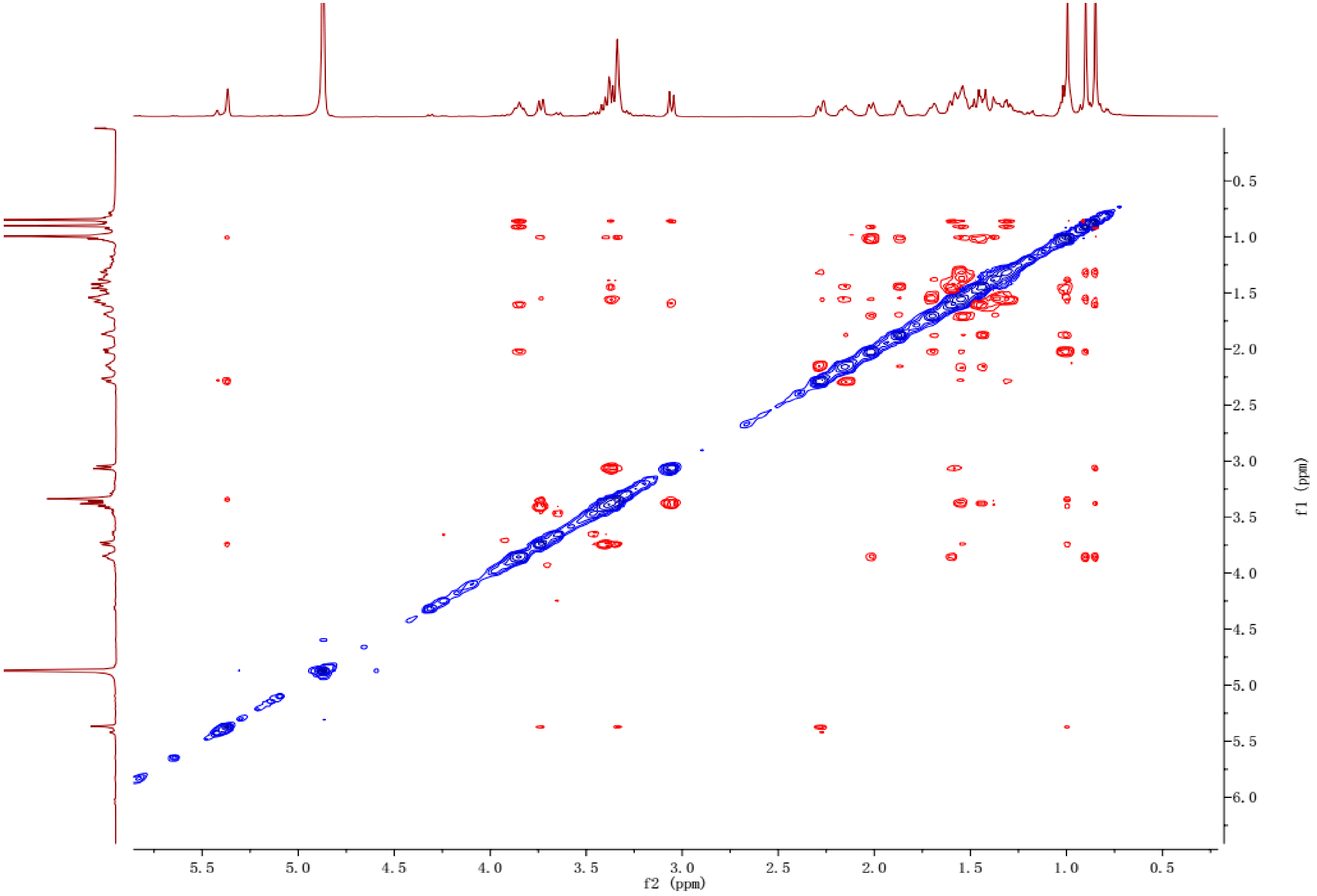

Figure S19. NOESY spectrum of compound **3** in CD_3OD.

Qualitative Analysis Report

**Data Filename**	20160815_LCMS_YY_ZYD_161289.d	**Sample Name**	PN-1B10A3
**Sample Type**	Sample	**Position**	P1-F1
**Instrument Name**	Agilent G6520 Q-TOF	**Acq Method**	20160516_LCMS_POS_5-95%.m
**Acquired Time**	8/15/2016 4:40:01 PM	**IRM Calibration Status**	Success
**DA Method**	small molecular data analysis method.m	**Comment**	LCMS

User Spectra

**Spectrum Source**	**Fragmentor Voltage**	**Collision Energy**	**Ionization Mode**
	175	0	ESI

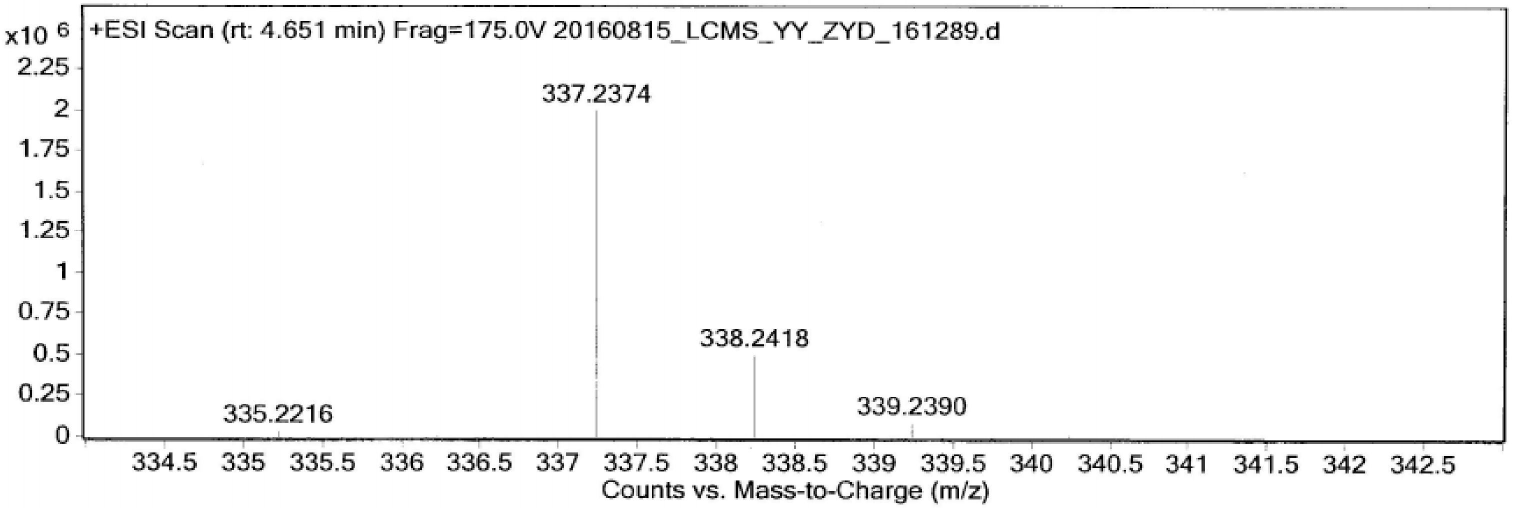

Formula Calculator Results

m/z	Calc m/z	Diff (mDa)	Diff (ppm)	Ion Formula	Ion
337.2374	337.2373	-0.07	-0.21	C20 H33 O4	(M+H)+

--- End Of Report ---

Figure S20. HR-ESI mass spectrum of compound **4**.

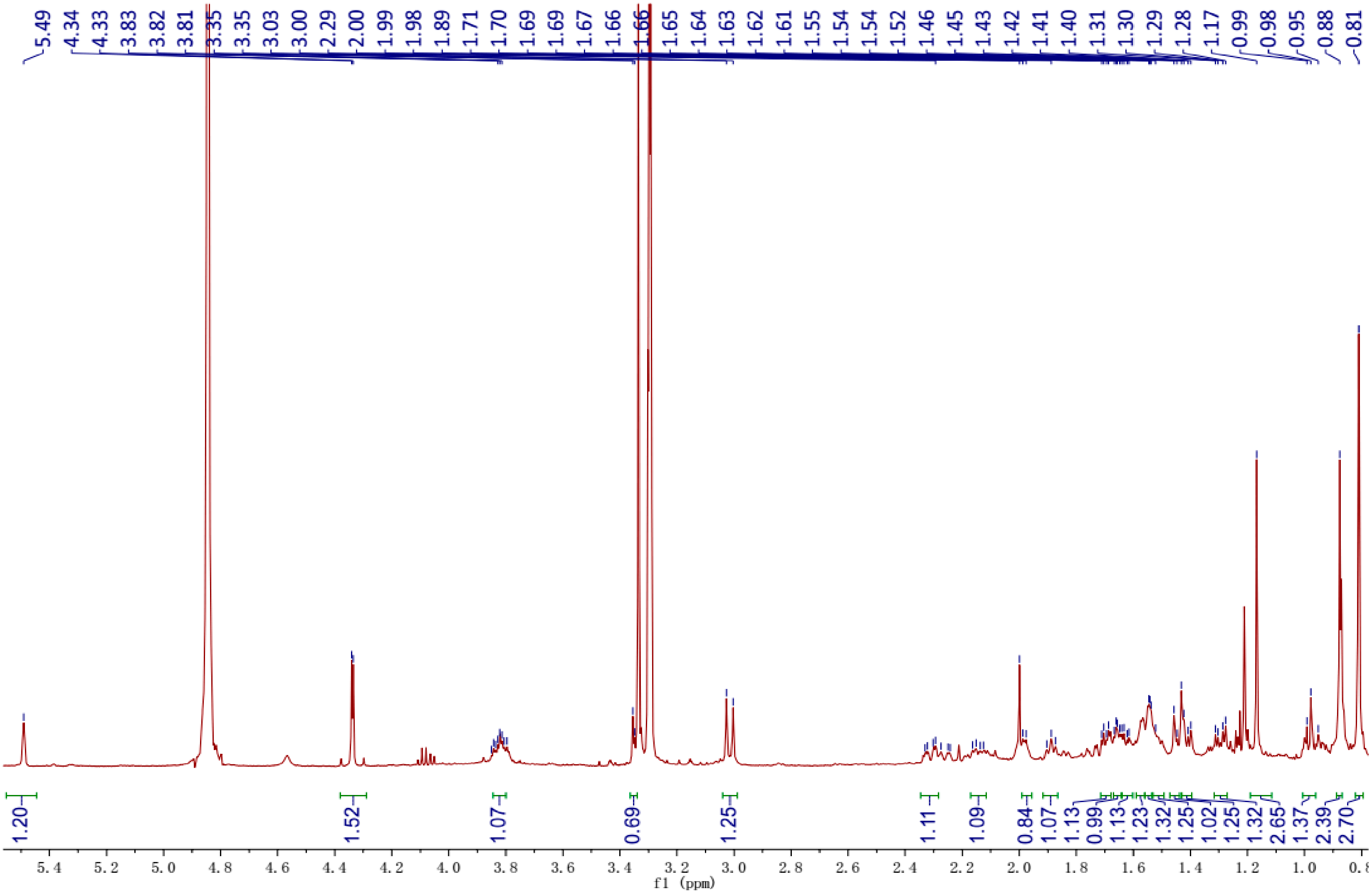

Figure S21. ^{1}H-NMR spectrum of compound **4** in CD_3OD (500 MHz).

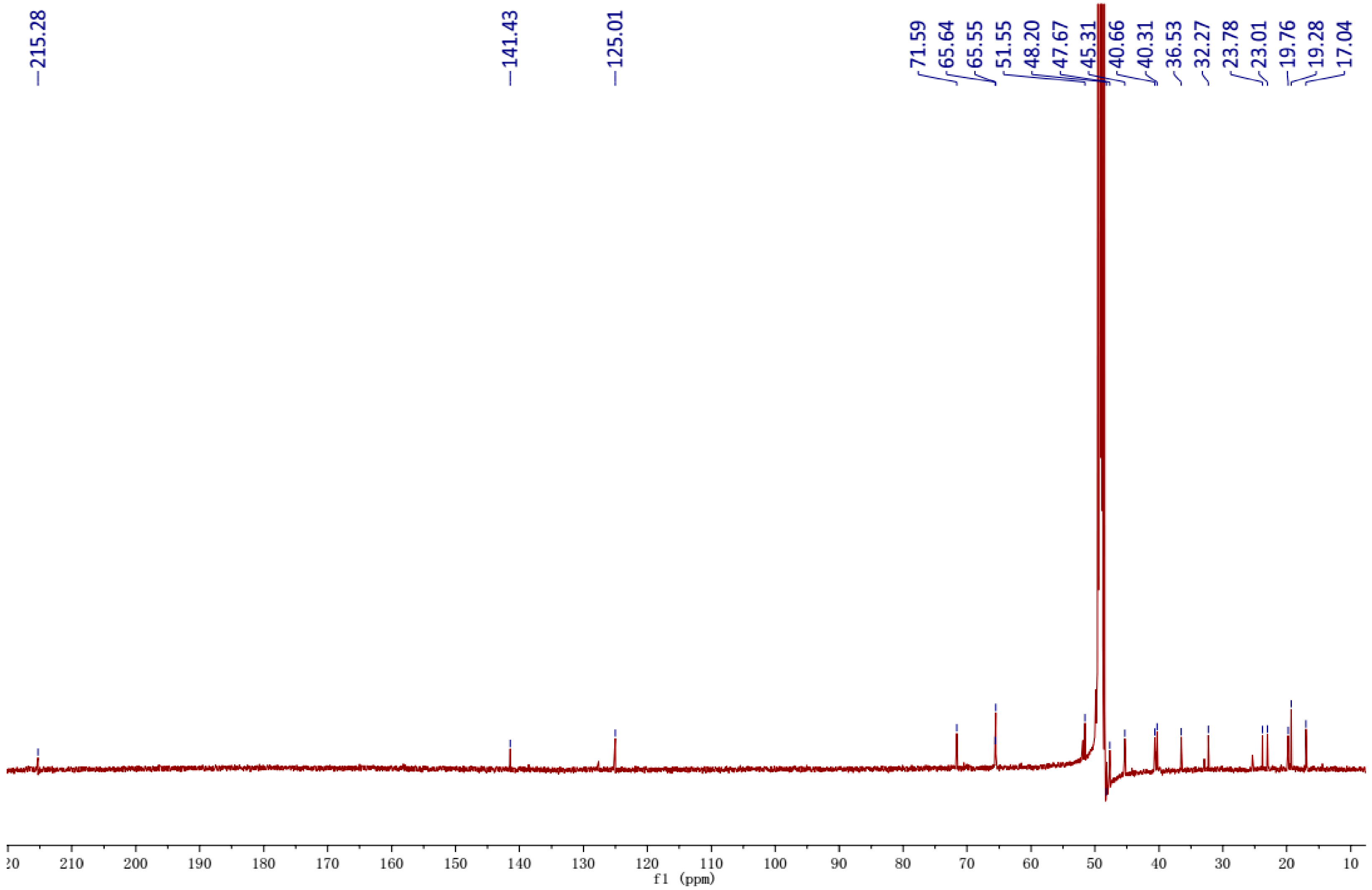

Figure S22. ^{13}C-NMR spectrum of compound **4** in CD_3OD (125 MHz).

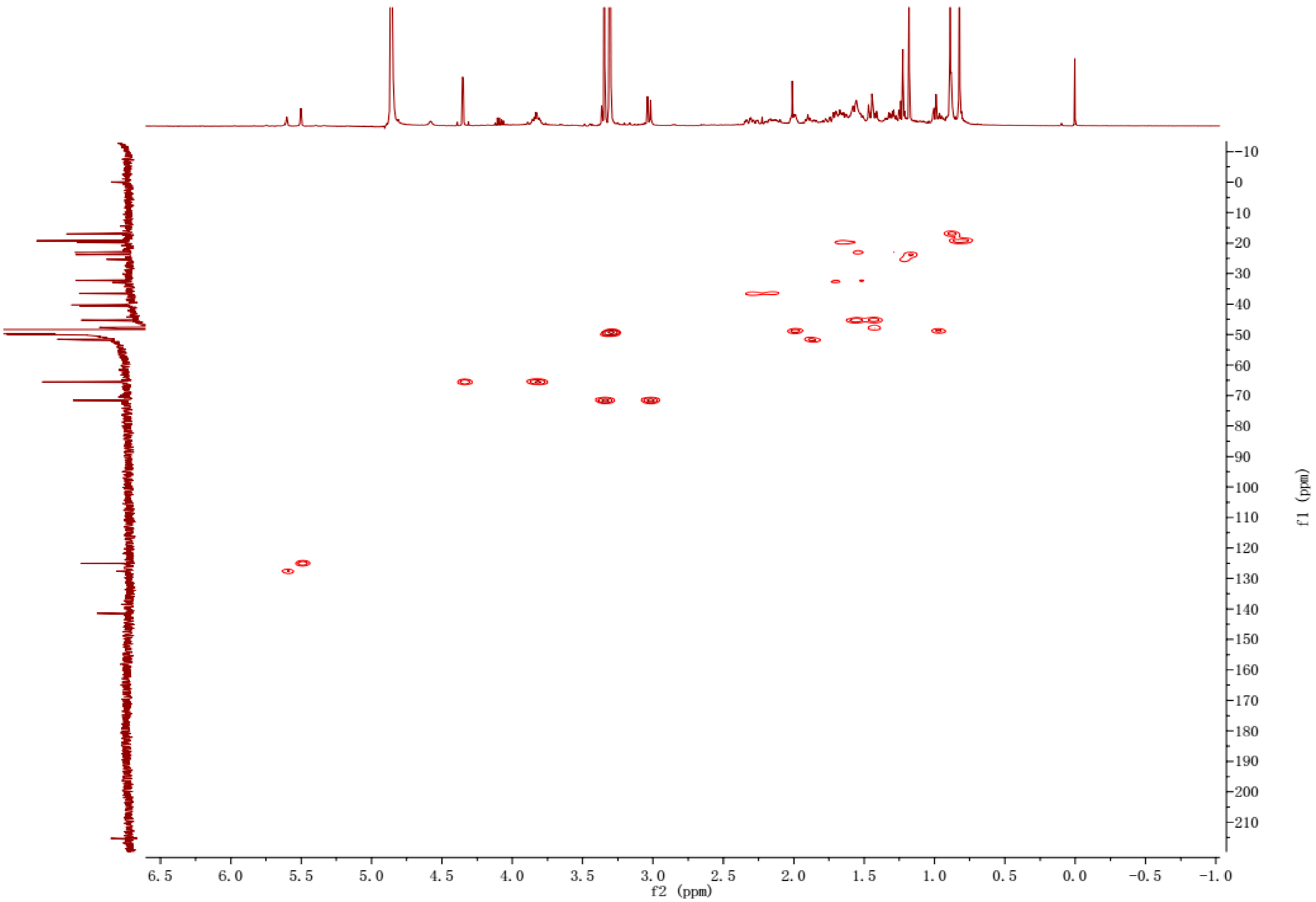

Figure S23. HSQC spectrum of compound **4** in CD_3OD.

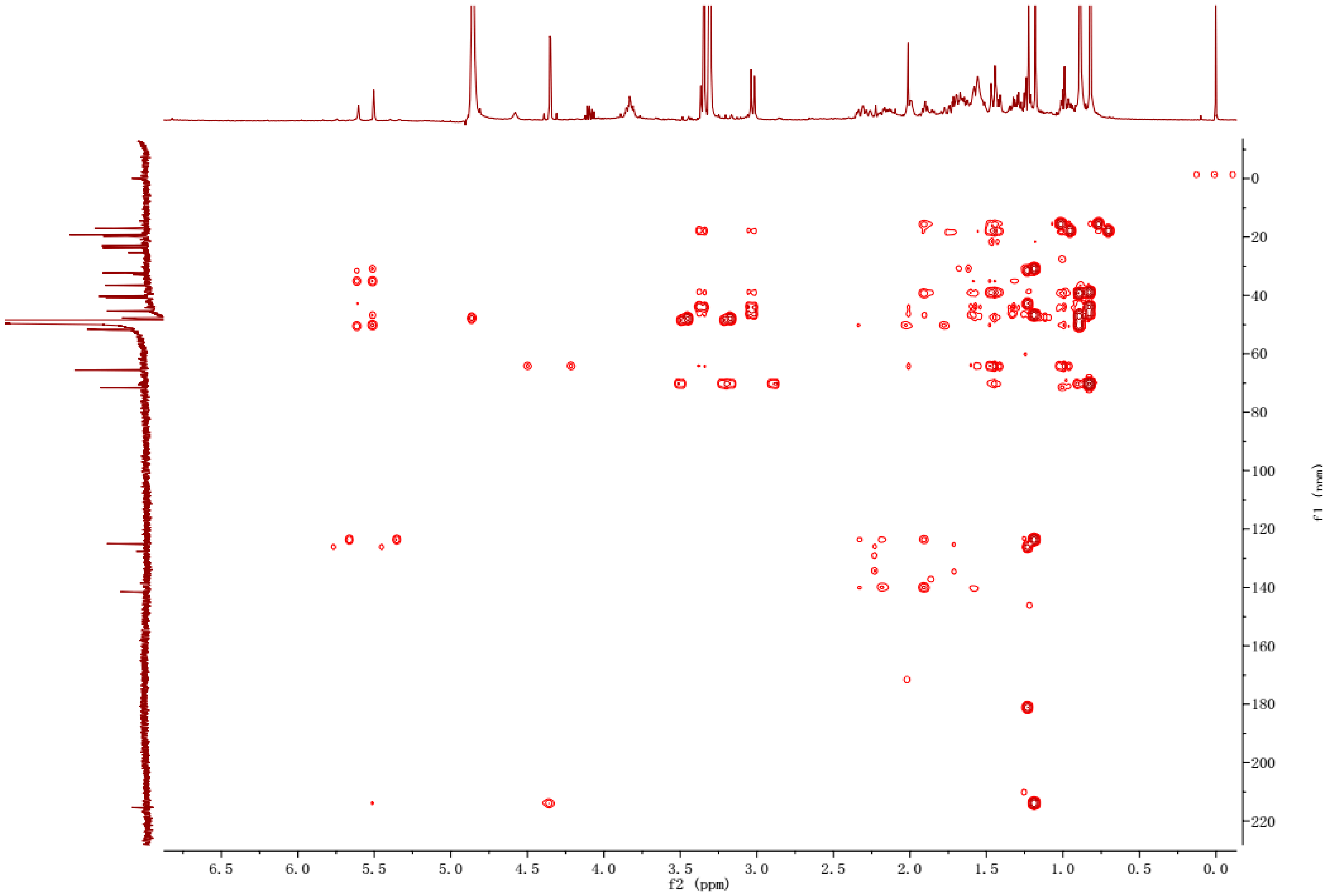

Figure S24. HMBC spectrum of compound **4** in CD_3OD.

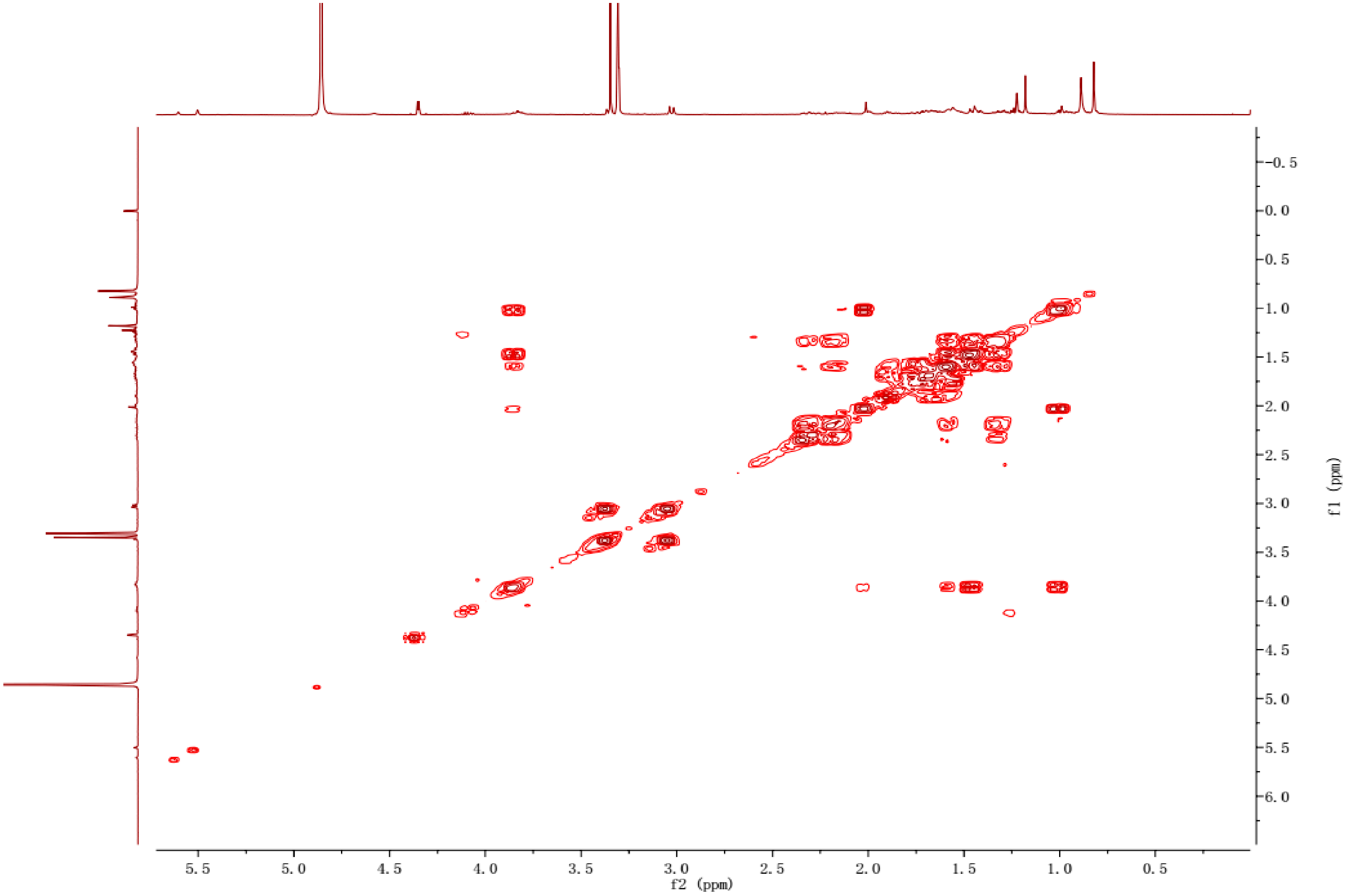

Figure S25. ^{1}H-^{1}H COSY spectrum of compound **4** in CD_3OD.

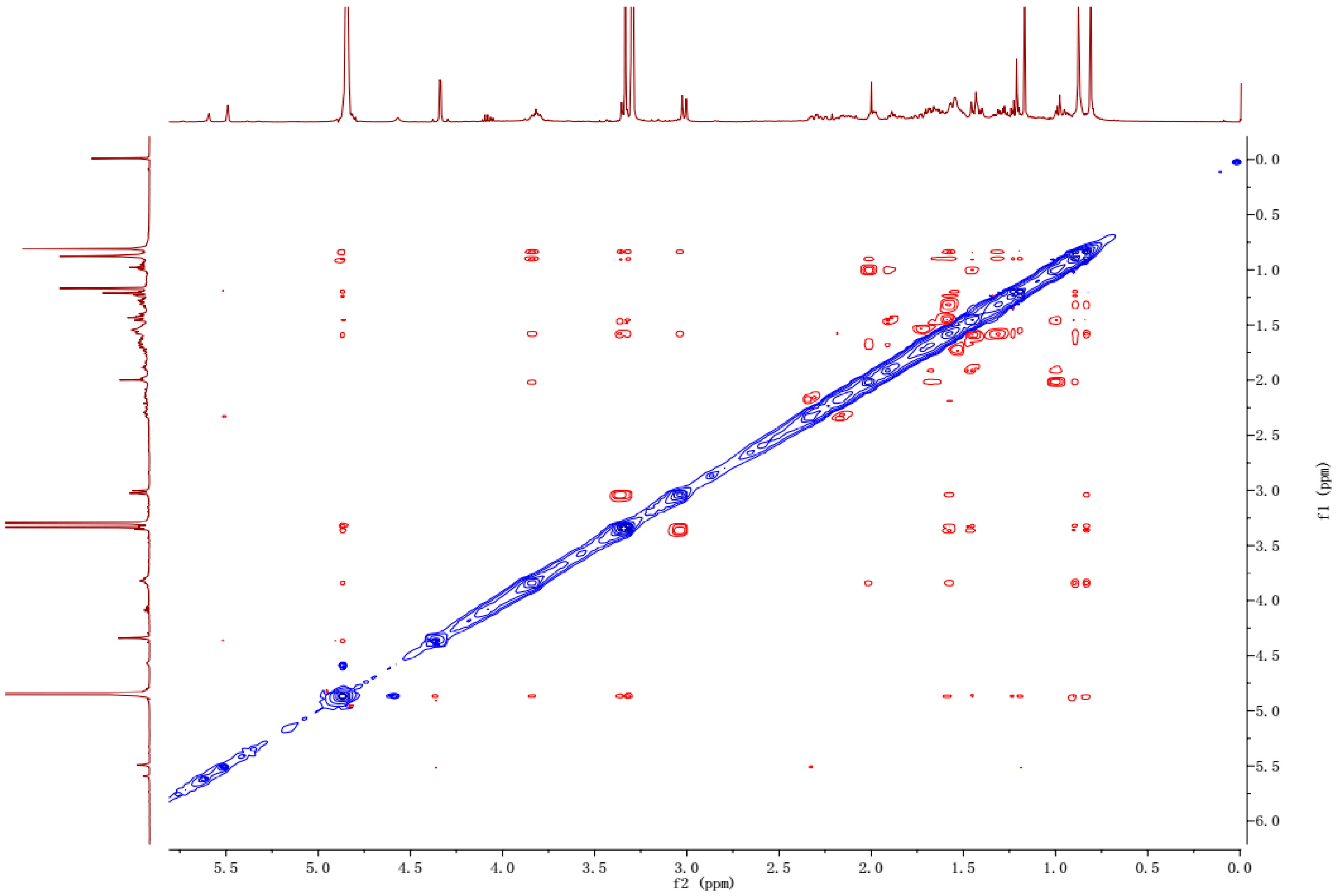

Figure S26. NOESY spectrum of compound **4** in CD_3OD.

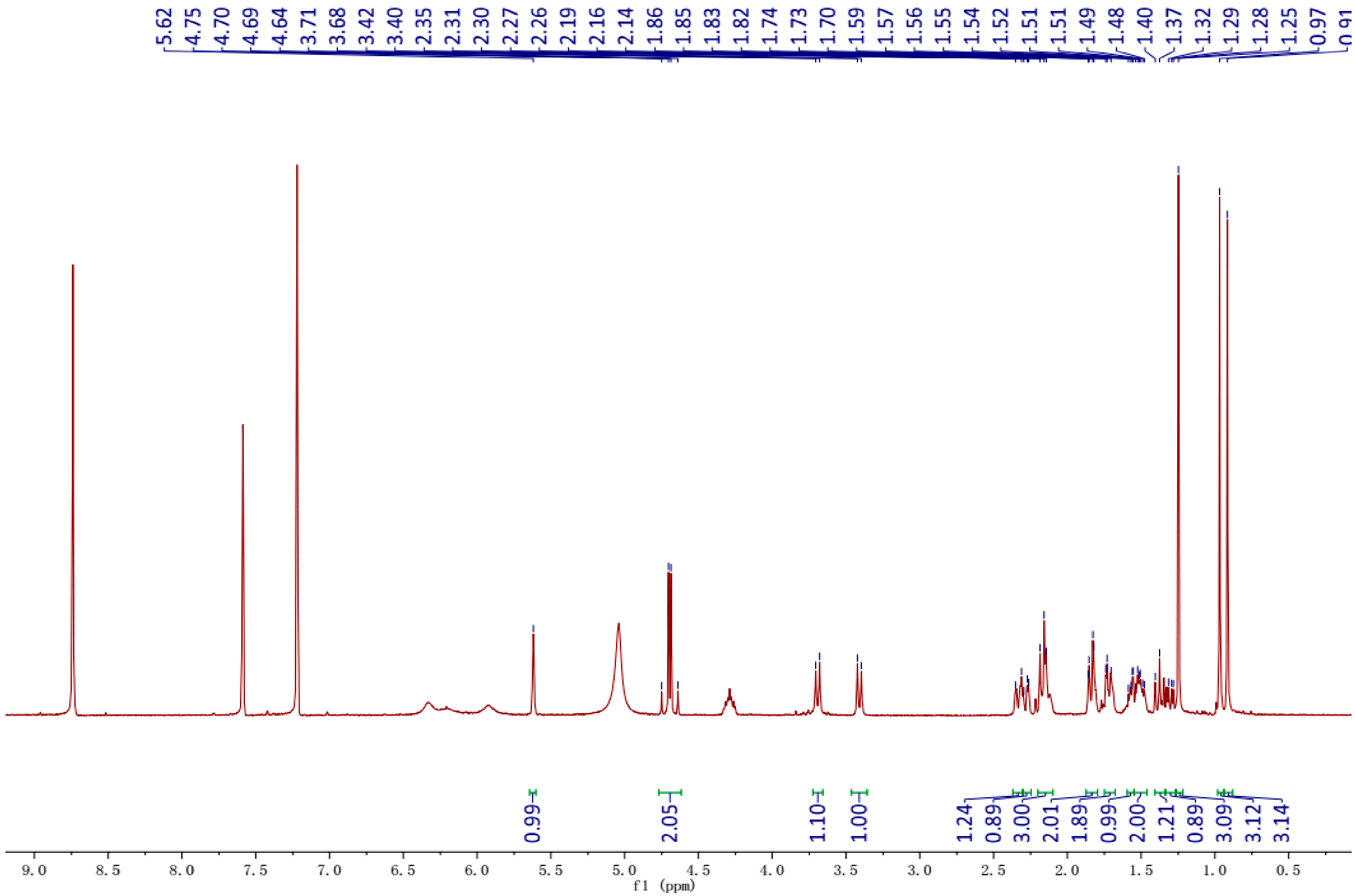

Figure S27. ^{1}H-NMR spectrum of compound **4** in Pyrdine-*d*$_5$ (400 MHz).

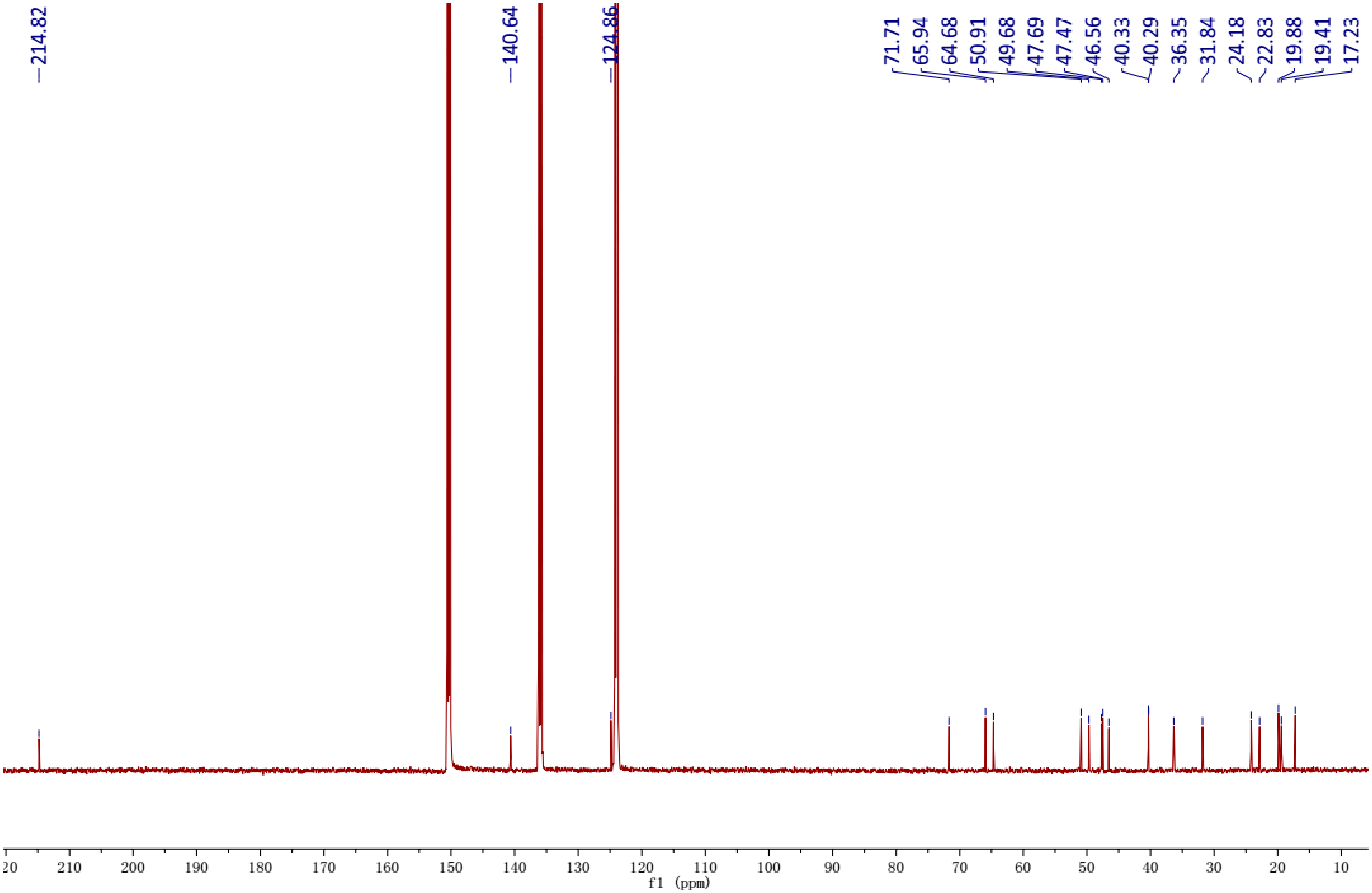

Figure S28. ^{13}C-NMR spectrum of compound **4** in Pyrdine-*d*5 (500 MHz).
